# From Sea to Therapy: Development and Analytical Control of Recombinant Human CDKL5 Production in the Marine Bacterium *Pseudoalteromonas haloplanktis* TAC125

**DOI:** 10.3390/md24050151

**Published:** 2026-04-24

**Authors:** Andrea Coletti, Marzia Calvanese, Flora Cozzolino, Ilaria Iacobucci, Concetta Lauro, Angelica Severino, Maria Monti, Ermenegilda Parrilli, Maria Luisa Tutino

**Affiliations:** 1 Department of Chemical Sciences, University of Naples Federico II, Complesso Universitario Monte S. Angelo, Via Cintia 4, 80126 Naples, Italy; andrea.coletti@unina.it (A.C.); flora.cozzolino@unina.it (F.C.); ilaria.iacobucci@unina.it (I.I.); concetta.lauro@unina.it (C.L.); angelica.severino@unina.it (A.S.); montimar@unina.it (M.M.); ermenegilda.parrilli@unina.it (E.P.); 2 Istituto Nazionale Biostrutture e Biosistemi I.N.B.B., Via dei Carpegna, 19, 00165 Roma, Italy; 3 CEINGE Advanced Biotechnologies, Franco Salvatore, Via Gaetano Salvatore 486, 80145 Naples, Italy

**Keywords:** Antarctic marine bacteria, cell factory, difficult to express proteins, ELISA, hCDKL5, protein phosphorylation, *Pseudoalteromonas haloplanktis* TAC125, quality by design

## Abstract

Marine bacteria are increasingly explored as alternative microbial platforms for the production of high-value biopharmaceuticals. In this study, we investigate the Antarctic marine bacterium *Pseudoalteromonas haloplanktis* TAC125 (*Ph*TAC125), an unconventional host capable of yielding soluble and biologically active human cyclin-dependent kinase-like 5 (hCDKL5). This serine/threonine kinase plays a crucial role in neuronal development, and its deficiency causes CDKL5 Deficiency Disorder, a severe and currently untreatable neurodevelopmental disease. Recombinant production of hCDKL5 is a prerequisite for the development of enzyme replacement therapy; however, current manufacturing processes remain insufficient for industrial translation, particularly in terms of product quality and functional consistency. To address these limitations, we developed dedicated analytical strategies: protein accumulation was quantified using a customised sandwich Enzyme-Linked Immunosorbent Assay (ELISA) designed to selectively detect full-length hCDKL5, while protein functionality was assessed by mass spectrometry-based quantification of autophosphorylation, a critical determinant of kinase activation. These complementary tools were applied to characterise hCDKL5 production under different growth conditions. Overall, this work establishes an integrated analytical framework aligned with a Quality by Design approach, enabling the simultaneous assessment of yield, structural integrity, and functional activation, and providing a robust basis for rational process optimisation towards scalable hCDKL5 manufacturing.

## 1. Introduction

The growing industrial demand for novel and versatile microbial chassis has drawn the attention of the scientific community towards unique environmental habitats known for their high biodiversity [1]. The Antarctic sea is one of such sources of distinctive microorganisms that remain poorly characterised [2,3]. Among these, *Pseudoalteromonas haloplanktis* TAC125 (*Ph*TAC125) stands out as a notable model for its potential as a microbial cell factory [3]. Key features such as its high capacity for translation and fast growth rate at sub-zero temperatures, combined with its ability to adapt and grow in a wide temperature range of −2.5 °C to 30 °C, make it a valuable candidate for several biotechnological applications [3,4,5,6]. In particular, its cold-adapted physiology is advantageous for the production of difficult recombinant proteins. At low temperatures, protein-folding kinetics are slowed down, allowing more time for correct conformational structuring, reducing overall misfolding and the formation of aggregation-prone intermediate [3,5]. In this context, *Ph*TAC125 exhibits specific cellular adaptations that support protein synthesis and folding under cold conditions, including a relatively high number of rRNA genes and tRNA genes that, combined with the overexpression of the Trigger factor and other chaperones, contribute to efficient translation and enhance protein folding processes [7].

These unique traits have inspired the development of integrated strategies to harness the potential of *Ph*TAC125. A genome-scale metabolic model has underlined its core metabolic reactions and identified key nutrients essential for achieving high growth rates and biomass accumulation, thereby enabling the formulation of an optimised synthetic medium [8,9,10]. In parallel, rational approaches to streamline the host genetic background for recombinant production led to the development of the *Ph*TAC125 genomics mutants and the design of dedicated expression systems, significantly improving production yields [11,12,13,14]. All this makes *Ph*TAC125 an excellent candidate for the recombinant manufacturing of proteins that are typically difficult to produce in conventional hosts, as already demonstrated by the successful production of several eukaryotic ones [15]. The most remarkable advancement is the recombinant production of the human cyclin-dependent kinase-like 5 (hCDKL5) [16]. hCDKL5 is a human serine/threonine kinase that has a key role in neuron development and synaptic activity [17,18,19]. Mutations in the gene coding for hCDKL5 are causative of CDKL5 Deficiency Disorder (CDD). CDD is a severe neurodevelopmental disorder that manifests from birth and currently lacks effective treatments [20]. Although a study on a mouse model demonstrated the feasibility of developing an enzymatic replacement therapy (ERT) by delivering the functional hCDKL5 to neuronal cells, significant challenges persist [21]. A fundamental requirement for clinical development is establishing a robust manufacturing process capable of yielding pure, biologically active, full-length hCDKL5. This task is particularly challenging due to the structural features of the protein, which include an extensive intrinsically disordered region (IDR) spanning nearly two-thirds of its sequence and a structured catalytic domain at the N-terminus [22,23]. Both the kinase domain and IDR domain contribute to making recombinant production of hCDKL5 highly susceptible to proteolytic degradation and aggregation, complicating efforts to maintain protein integrity [24,25]. Specifically, the kinase domain likely depends on specialised eukaryotic chaperone systems, such as the Hsp90-Cdc37 complex, for proper folding; accordingly, heterologous expression in bacterial hosts lacking this dedicated system can promote misfolding and the accumulation of inactive aggregates [25]. This instability is further exacerbated by the extensive IDR, which is inherently prone to proteolytic cleavage due to its disordered nature and high accessibility to cellular proteases.

Recombinant production of full-length hCDKL5 and truncated catalytic domain was attempted with several hosts, such as *E. coli* strains, yeast, insects, and HEK cell lines [22,26,27], but the recovered protein is mostly insoluble or in an aggregated state [25]. *Ph*TAC125 represents the first bacterial host allowing the recombinant production of soluble, active, full-length CDKL5, carried out with the specific intent to reach an industrial-level application [16]. Yet, the current process remains far from the level of industrial scale and requires significant intensification [16,28]. Achieving high titres while preserving structural and functional integrity is therefore a mandatory objective. Nowadays, the benchmark for the optimisation of novel drug production systems is the Quality by Design (QbD) approach [29]. Indeed, the QbD strategy allows the fine-tuning of any pharmaceutical production by looking at the whole process instead of the single steps. Applying QbD necessitates the development of dedicated analytical tools to monitor key quality parameters of the recombinant product [30]. For instance, the achieved recombinant hCDKL5 titres have been estimated by means of Western blot [28]. However, this methodology is affected by analytical limitations (i.e., including a relatively limited dynamic range, the need to identify the linearity range at which quantification is reliable) and technical limitations, such as the analysis of only denatured samples [31]. A possible means to overcome such a limitation, but still exploit the advantages of an immunoassay, is the Enzyme-Linked Immunosorbent Assay (ELISA). Different designs of ELISA can be applied for different analytical purposes. Each design of assay varies by means of recognising the analyte, type, and number of antibodies [32,33]. Among these formats, the sandwich ELISAs are particularly versatile in the interaction between the antibodies and the antigen. Since signal generation depends on the simultaneous recognition of the analyte by two antibodies [33], the careful selection of antibody pairs allows the acquisition of specific and detailed information about the target analyte. Despite the broad availability of commercial ELISA kits for many proteins, no such assay currently exists for hCDKL5. Therefore, one of the aims of this study is to harness the flexibility of the sandwich ELISA to develop a dedicated quantitative assay for full-length hCDKL5 recovered from *Ph*TAC125 bacterial lysate. This strategy enables the direct measurement of soluble recombinant hCDKL5, avoiding protein denaturation steps that may compromise both yield estimation and functional integrity [33]. The approach described herein is specifically designed to ensure selectivity towards the intact protein by employing two distinct immunoglobulins directed against the N- and C-terminal tags fused to hCDKL5. This dual-recognition system guarantees that only the full-length recombinant protein is detected, overcoming a major limitation of conventional quantification methods that are unable to discriminate between truncated or degraded species. Furthermore, to strengthen the analysis of both quality and quantity of the recovered product, the ELISA-based quantification is associated with a targeted mass spectrometry (MS) workflow. In particular, hCDKL5 enzymatic activity is known to be tightly regulated by the autophosphorylation of the TEY activation loop, a post-translational modification that plays a pivotal role in kinase function [27,34]. By leveraging MS, the phosphorylation status of key residues within this activation loop can be quantitatively assessed under the tested production conditions, offering a functional readout that goes beyond mere protein abundance.

Here, LC–MS/MS phospho-analysis is applied to compare hCDKL5 produced under different growth conditions, extending its use from phosphosite characterisation to product quality assessment. Comparisons are conducted at condition-specific optimised expression times (24 h for the reach medium and 5 h for synthetic media), reflecting the distinct expression kinetics of each medium and enabling evaluation under representative production settings [25]. The combined application of quantitative sandwich ELISA and MS-based characterisation thus represents a highly innovative and integrated analytical platform for recombinant hCDKL5 production. This dual approach enables simultaneous monitoring of protein yield, structural integrity, and functional phosphorylation state, providing a comprehensive quality profile of the recombinant product. Applying these complementary techniques as tools for process optimisation and intensification is fully aligned with a Quality by Design (QbD) framework, facilitating the development of a robust, reproducible, and industrially relevant manufacturing process for hCDKL5.

## 2. Results

### 2.1. Development of a Sandwich ELISA for Recombinant hCDKL5 Analysis

#### 2.1.1. Sandwich ELISA Design and Antibody Selection

**Figure 1:**
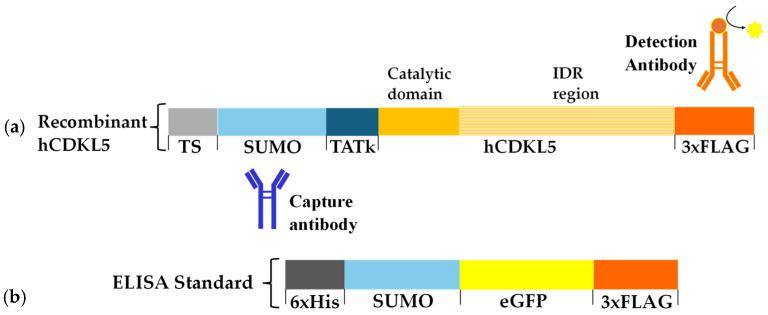
Schematic representation of the target and standard proteins of the sandwich ELISA. (**a**) Recombinant hCDKL5 construct with additional tags (not to scale) and ELISA antibodies’ binding sites. From N-terminus to C-terminus: (1) the Twin-Strep tag (TS) (grey); (2) the SUMO solubility tag (SUMO tag) (light blue); (3) the cell-penetrating peptide TATk (dark blue); (4) hCDKL5 protein (yellow). The structured catalytic domain is indicated by a full yellow box, while the intrinsically disordered region (IDR) is indicated by a yellow parallel-lines box; (5) A 3xFLAG tag (orange). The capture and detection antibodies used in the ELISA are represented, respectively, in blue and orange. (**b**) Schematic representation of the ELISA standard protein 6xHis-SUMO-eGFP-3xFLAG, showing the additional tags added to the protein sequence. From N-terminus to C-terminus: the 6xHis-tag sequence (6xHis) (grey); the SUMO-tag (light blue); the eGFP protein (yellow); a 3xFLAG tag is represented in orange. Boxes are not to scale.

A major bottleneck in optimising hCDKL5 production in the Antarctic bacterium is the lack of a dedicated analytical method to accurately quantify recombinant protein levels. This challenge is further exacerbated by the presence of multiple proteolytic fragments, which hinder the reliable quantification of full-length hCDKL5. To overcome these challenges, a sandwich ELISA that specifically recognises the N- and C-termini domains of the recombinant hCDKL5 was developed. To do so, the structure of the recombinant hCDKL5 construct used in this study was analysed to identify the optimal binding sites. The recombinant hCDKL5 construct incorporates multiple functional tags designed to enhance protein solubility, facilitate purification, and enable intracellular delivery. A Twin-Strep (TS) affinity tag is located at the N-terminus, followed by a SUMO tag, which improves protein solubility and provides a specific protease cleavage site for tag removal during downstream processing [35]. Immediately downstream of the SUMO tag, a TATk signal peptide is included to promote intracellular delivery of the recombinant enzyme and the crossing of the blood–brain barrier upon an intravenous administration of the protein [21]. A 3XFLAG tag is positioned at the C-terminus to enable additional affinity-based detection and purification. A schematic representation of the recombinant hCDKL5 construct is shown in Figure 1a (not to scale), highlighting its main structural features: (i) a structured N-terminal catalytic domain (full yellow box) and (ii) a large intrinsically disordered region (IDR) encompassing approximately two-thirds of the protein length (yellow parallel lines box) [22,23]. For the sandwich ELISA development, the N-terminal SUMO tag and the C-terminal 3xFLAG tag (Figure 1a) were selected as targets for the antibodies exploited in the assay. An anti-SUMO immunoglobulin is used as the capture antibody, which is immobilised on the microplate surface and selectively binds the protein species retaining an intact catalytic domain. The detection was performed using an anti-FLAG antibody conjugated to horseradish peroxidase (HRP), which targets the C-terminal 3xFLAG epitope to confirm the presence of the IDR region (Figure 1a). By requiring the simultaneous presence of both affinity tags, this configuration enabled the selective detection of full-length hCDKL5 while excluding truncated or partially degraded variants, and providing a platform for subsequent quantitative analyses.

#### 2.1.2. Standard Protein Design and Assay Development

**Figure 2:**
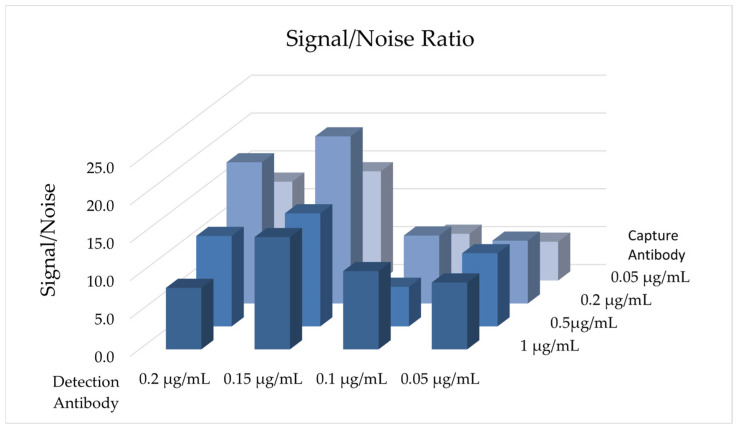
Identification of the optimal sandwich ELISA conditions based on signal-to-noise ratio. Four concentrations of capture (1, 0.5, 0.2, 0.05 μg/mL) and detection (0.2, 0.15, 0.1, 0.05 μg/mL) antibodies were tested, resulting in 16 possible capture/detection combinations. Each condition was evaluated with 2 nM of the standard protein. The signal-to-noise ratio was calculated as the ratio of absorbance measured in the presence of standard protein to the background signal measured in its absence. In the column graph, the detection and capture antibody concentrations are displayed on the X and Y axes, respectively. On the *Z*-axis, the signal/noise ratio for each condition is reported.

**Figure A2:**
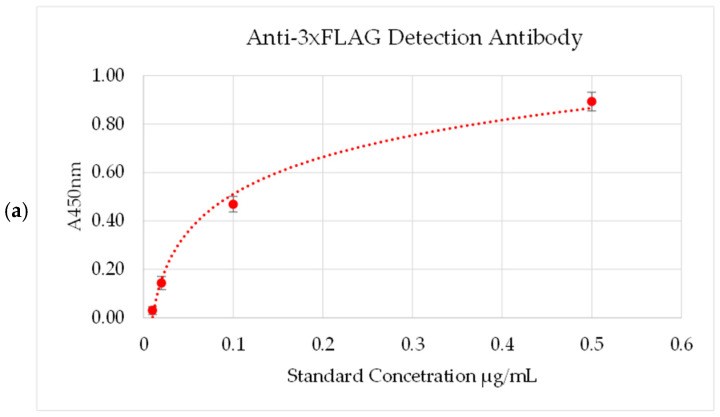

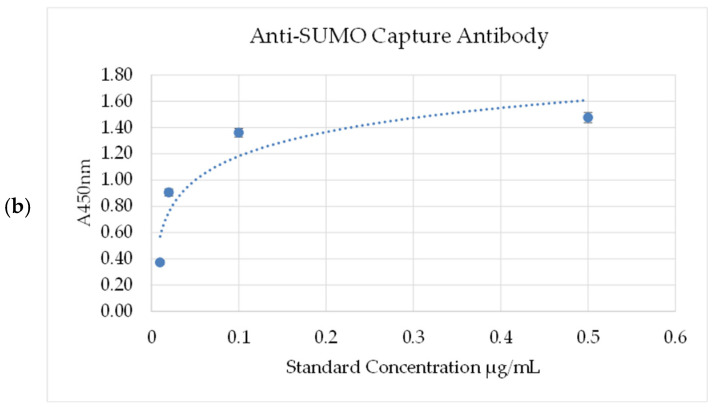
Direct and indirect ELISA on standard protein 6xHis-SUMO-eGFP-3xFLAG reactivity with anti-SUMO and anti-Flag antibodies. The red chart (**a**) reports output data from the detection antibody test. The blue chart (**b**) reports output data from the capture antibody test. The antibodies exploited for the sandwich ELISA were anti-SUMO antibody and anti-3xFLAG HRP-conjugated antibody. The first was exploited as a capture antibody, the latter as a detection antibody. To test the reactivity of 6xHis-SUMO-eGFP-3xFLAG with each antibody, two ELISA assays were carried out. Two MaxiSorp multiwell-96 plates were coated overnight at 4 °C with four dilutions of purified 6xHis-SUMO-eGFP-3xFLAG (10.2 nM, 2.0 nM, 1.0 nM, and 0.4 nM) in PBS 1X. The same volume of 100 μL was applied for each concentration. Then, the plates were washed with 300 μL/well of PBS-Tween 0.05%*v*/*v* (PBST) three times. A blocking step was performed with 300 μL of blocking buffer (PBST + 3% *w*/*v* BSA) for 2h at 37 °C to avoid nonspecific binding of the detection antibody to the well surface. Then, one plate was incubated with 0.1 μg/mL of the anti-Flag HRP-conjugated detection antibody. The latter plate was incubated with 0.1 μg/mL of anti-SUMO capture antibody. Both plates were left at 25 °C for 1 h. Plates were washed with PBST three times. Hence, the plate with anti-SUMO antibody was incubated with the anti-Mouse HRP-conjugated antibody (Sigma) for 1 h at 25 °C. The anti-Mouse antibody was 1:10′000 diluted with the blocking buffer. One last wash step was performed with PBST for this plate. Lastly, 100 μL (TMB) was added to each well and incubated for 10′. Hence, the reaction was stopped with 0.18M sulphuric acid (100 μL/well). Signal detection was carried out with a plate reader measuring A450 nm.

**Figure A3:**
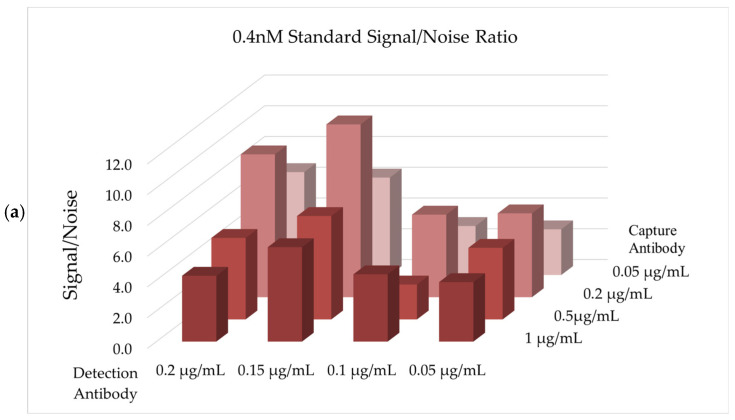

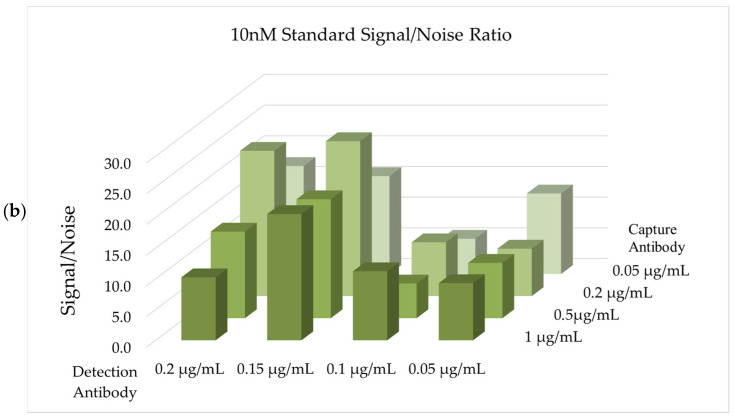
Identification of the optimal sandwich ELISA conditions based on signal-to-noise ratio with standard protein. Four concentrations were tested for each antibody (capture antibody: 1 μg/mL; 0.5 μg/mL; 0.2 μg/mL; 0.05 μg/mL; detection antibody: 0.2 μg/mL; 0.15 μg/mL; 0.1 μg/mL; 0.05 μg/mL) for a total of 16 possible combinations of capture and detection antibodies. Each condition was tested with three different standard protein concentrations (10.2 nM, 2.0 nM, and 0.4 nM). The ELISA plate was previously derivatised with the capture antibody and blocked with PBS-Tween 0.05% + 3% BSA. Standard protein was incubated for 18h at 4 °C. Detection antibody was added in each well after the wash step with PBS-Tween 0.05%. Signals were developed by TMB incubation, and A_450nm_ was measured after stopping the reaction with 0.18M sulphuric acid. For each condition, the signal/noise ratio was calculated by dividing the A_450nm_ detected with the standard protein by the A_450nm_ measured with 0 μg/mL of standard protein. The experiment was carried out with three different standard protein concentrations: 500 μg/mL (**b**), 100 μg/mL (Figure 3), and 20 μg/mL (**a**). In the column graph, the detection and capture antibody concentrations are displayed on the X and Y axes, respectively. On the *Z*-axis, the signal/noise ratio for each condition is reported.

To establish the sandwich ELISA as a quantitative analytical tool, a robust and reproducible calibration curve was essential. However, the absence of a pure, stable, and full-length hCDKL5 preparation precluded its direct use as a calibration standard. To overcome this limitation, a dedicated recombinant standard protein was rationally designed. The recombinant 6xHis-SUMO-eGFP-3xFLAG (Figure 1b) protein was chosen as the standard because it replicates the epitope architecture exploited by the assay without the structural complexity of hCDKL5. Indeed, this construct contains the SUMO tag at the N-terminus and the 3xFLAG tag at the C-terminus, which are specifically recognised by the capture and detection antibodies, respectively. The eGFP protein included between the two epitopes acts as a structured spacer, thereby minimising steric hindrance and preventing competitive antibody interactions during sandwich formation [36]. Such a protein was recombinantly produced in *Escherichia coli* BL21 (DE3) and subsequently purified as described in the Materials and Methods Section 5.2.1 and Section 5.3. Before implementing the sandwich configuration, the performances of capture and detection antibodies were independently validated using indirect and direct ELISA configurations, respectively. These preliminary tests confirmed the accessibility and immunoreactivity of both epitopes (Appendix A, Figure A2). Subsequently, the sandwich ELISA conditions were optimised by testing four different concentrations of both capture and detection antibodies. For the capture antibody, concentrations of 1, 0.5, 0.2, and 0.05 μg/mL were evaluated to identify the condition that ensures the complete saturation of the plate wells [37]. Instead, detection antibody concentrations (0.2, 0.15, 0.1, and 0.05 μg/mL) were selected according to the manufacturer’s instructions. Each antibody combination was tested with three concentrations of the standard protein (10 nM, 2 nM, and 0.4 nM) to evaluate linearity and dynamic range. Assay performance was assessed based on selectivity and signal output for the target molecule, and the optimal configuration was defined by the highest signal-to-noise ratio, calculated as the ratio of absorbance measured in the presence of standard protein to the background signal measured in its absence. As shown in Figure 2, the highest signal-to-noise ratio was achieved using 0.2 μg/mL of capture antibody and 0.15 μg/mL of detection antibody with 2 nM of standard protein, whereas the results obtained at the other tested standard concentrations are reported in Appendix A (Figure A3a,b).

**Figure 3:**
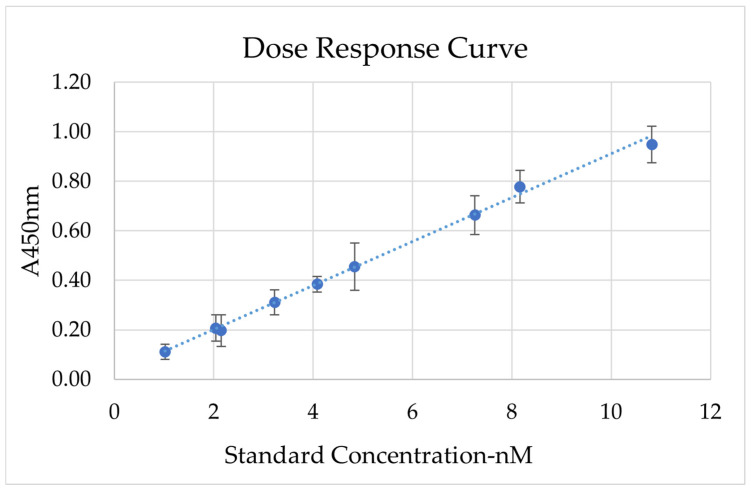
Dose–response curve of the standard protein in the sandwich ELISA assay condition identified from signal/noise screening. The chart shows the average dose–response curve obtained from five independent replicas. The top graph shows the average curve obtained, where y = 0.089x + 0.023, R^2^ 0.96, and *p*-value < 0.01. The *X*-axis represents the nM concentration, while the *Y*-axis displays the absorbance at 450 nm for each calibration point. ELISA data were analysed with the regression method and ANOVA analysis. LOD of 00.84 nM and LOQ of 2.54 nM were estimated with the residual method [38].

**Figure A4:**
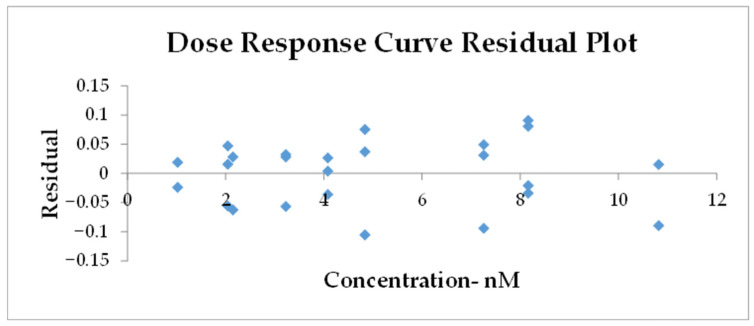
Dose–response curve residual plots. The chart below shows the calculated residual plot, comparing predicted data with true measurements. The *X*-axis represents the nM concentration, while the *Y*-axis displays the absorbance at 450 nm for each calibration point.

Once assay conditions were established, the linear range of the ELISA was determined by testing serial dilutions of the standard, ranging from 61 nM to 0.6 nM. A calibration curve was built with dilution points that reflected a linear reduction in the signal. In Figure 3, the average calibration curve built from five independent replicas is reported (Appendix A, Table A1). The curve was obtained by focusing on the concentration range of 1–11 mM due to the saturated signal obtained at higher concentrations. Regression analysis was coupled with residual analysis and a *t*-test to assess the statistical robustness of the assay curve (Appendix A, Figure A4). The final plot displays a narrow dynamic linear range in which the quantification is achievable (Appendix A.2 ELISA calibration curves). Furthermore, to fully characterise the developed assay, the Limit of Detection (LOD) and Limit of Quantification (LOQ) were calculated from the average dose–response using Formulas (1) and (2) [38]. An LOD of 0.84 nM and an LOQ of 2.54 nM were measured. Hence, for CDKL5 quantification in soluble lysate, a wide range of dilution factors has to be tested for the quantification to be accurate and fall within the assay linearity range and above the LOQ.

### 2.2. Impact of Media Composition on Recombinant hCDKL5 Production in PhTAC125

#### 2.2.1. hCDKL5 Recombinant Production in PhTAC125

The recombinant production of full-length hCDKL5 in *Ph*TAC125 has been previously established, demonstrating the ability of this psychrophilic host to produce soluble and catalytically active forms of this structurally complex protein [16,39]. However, the current laboratory-scale production process requires further optimisation to support scale-up and industrial feasibility.

The above-described recombinant construct, TS-SUMO-TATk-CDKL5-3xFLAG, is inserted into the psychrophilic expression vector pB40-BCD2 and expressed in the *Ph*TAC125 KrPl *LacY^+^* strain [16]. To assess the impact of culture medium composition on growth performance and recombinant protein production, recombinant *Ph*TAC125 was grown in three different media:

1. A rich medium based on yeast extract and peptone (TYP);
2. A synthetic medium based on Gluconate and Glutamate (GG) [9];
3. A modified version of the GG medium supplemented with maltose (GGM), as suggested by previous publication Fondi et al (2021) [28].

Cultivations were carried out at 15 °C, and recombinant protein production was induced with IPTG during the middle–late exponential growth phase (Supplementary Figure S1a–c). Based on previously optimised and standardised conditions for each medium, reflecting their distinct expression kinetics, cultures were harvested 5 h after induction in synthetic media [16] and 24 h after induction in TYP medium, a condition recently established in our laboratory. Comparative analysis of the three culture conditions revealed significant differences in growth kinetics and biomass accumulation (Table 1). Among the synthetic media, cultures grown in GGM reached higher final biomass concentrations and biomass volumetric productivity compared to GG. The maximum specific growth rate (µ_max_) observed in GGM (0.19 ± 0.02 h^−1^) was equal to that obtained in TYP (0.19 ± 0.02 h^−1^), whereas GG resulted in a slightly lower µ_max_ (0.13 ± 0.01 h^−1^). Despite having the same µ_max_ values, TYP-grown cultures achieved higher final biomass concentrations (C_bacterial_)_,_ biomass volumetric yield (Y_biomass_) and biomass volumetric productivity (Q_vbiomass_) than GGM.

**Figure 4:**
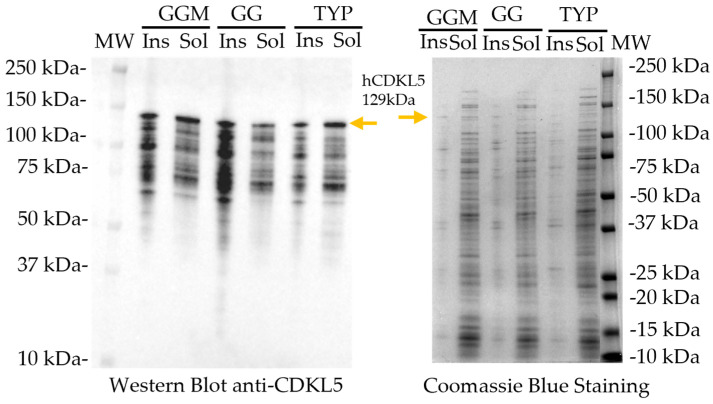
Solubility analysis of recombinant hCDKL5 expressed in *Ph*TAC125 KrPl *LacY*^+^ cells grown in three different media (GGM, GG, TYP). Soluble (Sol) and insoluble (Ins) fractions collected after centrifugation were analysed by SDS-PAGE (**right panel**) and Western blot using an anti-CDKL5 antibody (**left panel**). MW: Molecular weight (Precision Plus Protein Dual Colour Standards).

To assess the impact of medium composition on recombinant hCDKL5 production and solubility, soluble and insoluble fractions of *Ph*TAC125 recombinant cell lysates were analysed by SDS–PAGE followed by Western blot (Figure 4). Across all tested media, recombinant hCDKL5 was detected at the expected molecular weight of approximately 129 kDa, together with additional lower-molecular-weight bands consistent with partial protein degradation (anti-CDKL5 Western blot panel). Comparable signal intensities corresponding to the full-length protein were observed in the soluble fractions of cells grown in TYP and GGM media. By contrast, cultures grown in GG medium exhibited a markedly reduced accumulation of hCDKL5 in the soluble fraction, accompanied by an increased proportion of the protein in the insoluble fraction.

#### 2.2.2. Sandwich ELISA-Based Quantification of Recombinant hCDKL5 in Bacterial Lysates

**Figure 5:**
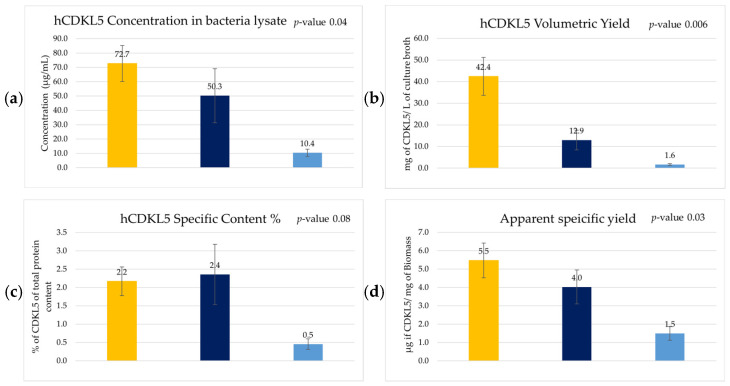
Quantitative analysis of recombinant hCDKL5 production in *Ph*TAC125 KrPl *LacY*^+^ cultures grown in TYP, GGM, and GG media by sandwich ELISA. Recombinant hCDKL5 quantification was performed, correlating the output signals to protein concentration using a standard calibration curve. Estimated production parameters for each media are reported: TYP (yellow); GGM (dark blue); GG (light blue): (**a**) Recombinant hCDKL5 concentration measured in the soluble bacterial lysate of *Ph*TAC125 KrPl *LacY*^+^. (**b**) Volumetric Yield for recombinant hCDKL5 in each growth condition. Amount of hCDKL5 recovered per litre of culture broth processed. (**c**) Specific content of hCDKL5 over the total protein content of the bacterial lysate. Total protein concentration of the lysate was estimated by means of Bradford analysis. (**d**) hCDKL5 apparent specific yield per biomass processed during lysis. All graphs display the arithmetic average of three replicas with the specific standard error. The *p*-value was estimated using single-factor ANOVA analysis.

**Figure A5:**
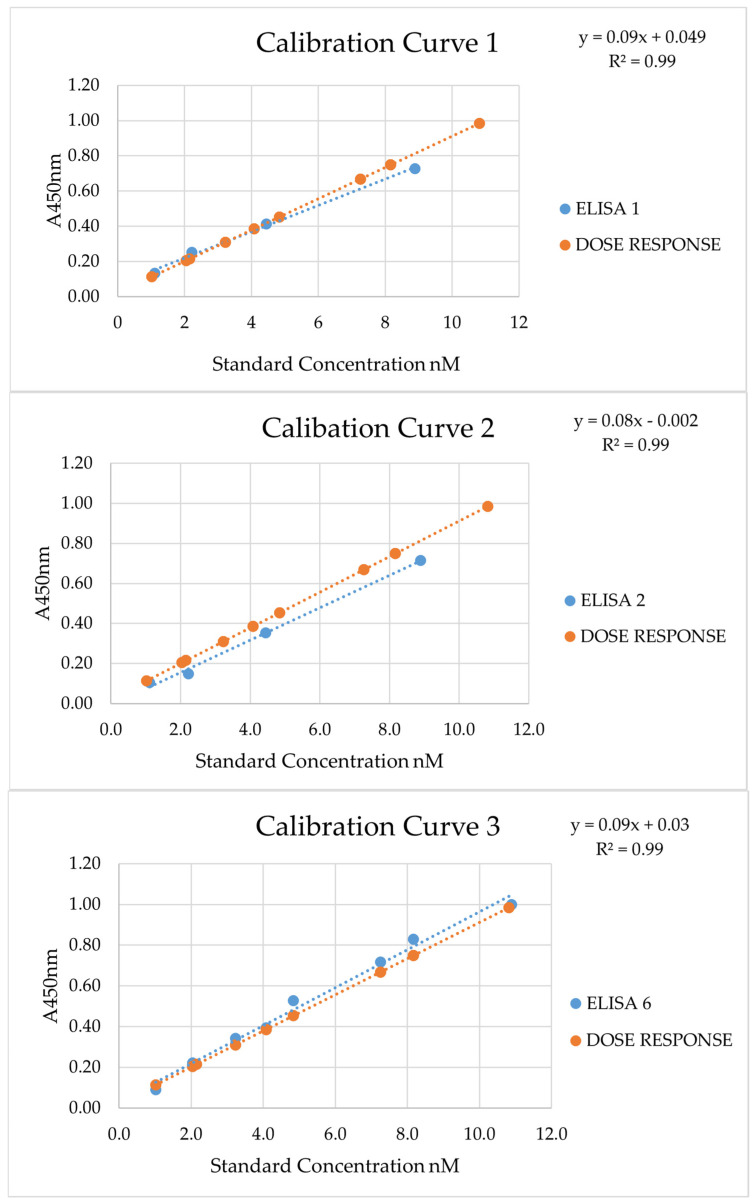
The 6xHis-SUMO-eGFP-3xFLAG calibration curves. The charts reported at the top show the calibration curves exploited for the quantification of hCDKL5 in each replica. The *X*-axis represents the nM concentration, while the *Y*-axis displays the absorbance at 450 nm for each calibration point. The calibration curve equations are reported in the top-right corner of each chart. In blue: the measured calibration curve; in orange: the average dose–response curve (master curve).

Before the analysis of bacterial lysates, potential matrix interferences related to the lysis buffer composition and sample dilution were evaluated. To this aim, known amounts of the standard protein were spiked into not-induced (NI) bacterial lysates, and the ELISA response was assessed. This preliminary analysis allowed the identification of sample dilution conditions that minimised matrix effects, ensuring that the measured signals fell within the linear range of the calibration curve and avoided the Hook Effect [40] (Appendix A, Table A1 and Table A2). For each sample, serial dilutions from 1:10 to 1:160 dilution factors were performed and analysed on an ELISA plate. Once the sandwich ELISA conditions and calibration curve were defined, soluble hCDKL5 levels in *Ph*TAC125 KrPl *LacY*^+^ bacterial lysates were quantified. Equal biomass amounts (30 OD_600nm_) from the different cultures were lysed. Quantitative analysis was performed by interpolation against the calibration curve, considering only dilution points within the linear dynamic range of the assay (Appendix A, Figure A5). As shown in Figure 5a, cultures grown in GG, the reference condition for recombinant hCDKL5 production [16], displayed low levels of soluble hCDKL5 (10.4 ± 2.5 µg/mL), confirming the limited production of this baseline condition. Supplementation of GG with maltose (GGM) resulted in a marked increase in soluble hCDKL5 concentration (50.3 ± 18.9 µg/mL), reaching values comparable to those obtained in the rich TYP medium (72.7 ± 12.5 µg/mL). A similar trend was observed when analysing hCDKL5 volumetric productivity (Q_VhCDKL5_) (Table 2). While GG cultures showed a low hCDKL5-specific content (0.5 ± 0.1% of total soluble protein content), maltose supplementation brought hCDKL5% to levels comparable to TYP, with values in the range of approximately 2.2–2.4% of total soluble protein content for both GGM and TYP conditions (Figure 5c). Consistently, GGM cultures exhibited a clear improvement over GG in the hCDKL5 apparent specific yield (Ys_hCDKL5_) (4.0 ± 0.9 µg/mg of biomass; Figure 5d), Volumetric Yield (Y_hCDKL5_) (12.9 ± 4.6 mg/L of culture broth; Figure 5b), and volumetric productivity (0.6 ± 0.2 mg/(L·h); Table 2). The apparent specific yield parameter is defined as the amount of hCDKL5 recovered in the soluble extract per mg of processed biomass following lysis buffer treatment. Despite the substantial enhancement achieved through maltose supplementation, TYP medium was more effective in terms of apparent specific yield (5.5 ± 0.9 µg/mg of biomass) and Volumetric Yield (42.4 ± 8.8 mg/L of culture broth) (Figure 5b–d), whereas metabolic optimisation of the synthetic GG medium could only partially recover production.

### 2.3. Mass Spectrometry-Based Analysis of Recombinant hCDKL5 Autophosphorylation State

Building upon our previously reported protocol for the investigation of hCDKL5 autophosphorylation, LC–MS/MS analysis was employed to compare the functional maturation of the full-length protein produced under different conditions [25]. Using GG medium as the baseline condition, the structural and functional characterisation of recombinant hCDKL5 was extended beyond yield and accumulation to include an analysis of its autophosphorylation status. Attention was given to the conserved TEY activation loop (residues 169–171), whose phosphorylation has been previously associated with CDKL5 activation [27,34]. To this end, mass spectrometry analyses were performed on enriched hCDKL5 produced under GG, GGM, and TYP culture conditions. To validate the origin of the detected phosphorylation events, a kinase-dead CDKL5 mutant (KK42–43RR variant), expressed in recombinant *Ph*TAC125 cells grown in TYP medium, was analysed in parallel as a negative control for autophosphorylation.

For all samples, enriched protein fractions were first resolved by SDS–PAGE, and bands corresponding to the expected molecular weight (129 kDa) of hCDKL5 were excised and subjected to in-gel tryptic digestion. Peptide mixtures were subsequently analysed by LC–MS/MS using a high-resolution Orbitrap Exploris 240 mass spectrometer. Peptide identification and phosphorylation site assignment were carried out with Mascot software, considering a +79.966 Da mass shift indicative of phosphate addition on Ser, Thr, or Tyr residues. All phosphorylation sites were further validated by manual inspection of the MS/MS spectra.

Overall sequence coverage of hCDKL5 in the range of 90–96%, ensuring the representation of both the catalytic domain and the intrinsically disordered region. The MS search strategy focused on phosphorylation events, with particular paid attention to Ser/Thr/Tyr residues and Tyr171-containing peptides. No additional post-translational modifications were confidently identified under the applied (phosphorylation-focused) search parameters. A dedicated LC–MS/MS analysis of possible post-translational modifications affecting the TATk peptide was not performed, as the workflow was specifically designed to investigate hCDKL5 phosphorylation within the catalytic region; accordingly, no modifications within the TATk region were confidently identified under the applied conditions. Phosphorylation introduces a mass increase of approximately +79.966 Da at the molecular level, whereas the corresponding shift observed in MS spectra is expressed in *m*/*z* and depends on the ion charge state; doubly and triply charged phosphorylated peptides show shifts of approximately 40 and 26.6 *m*/*z* units, respectively.

Phosphoproteomic analysis revealed multiple phosphorylation events on recombinant hCDKL5, including the modification of Tyr171 within the TEY motif. While phosphorylation of this residue was detected under all tested conditions, its relative abundance differed markedly depending on the culture medium. Quantitative analysis based on extracted ion chromatograms (XICs), comparing phosphorylated and unmodified peptide forms [25,41,42], showed the highest levels of Tyr171 phosphorylation in TYP-grown cells, intermediate levels in GGM, and the lowest abundance in GG. This approach enabled a relative quantification of phosphorylation levels by comparing phosphorylated and non-phosphorylated forms of the same peptide. Mascot analysis specifically confirmed the monophosphorylation of the tryptic peptide spanning residues 159–175 (NLSEGNNANYTEYVATR) at Tyr171 within the catalytic domain.

As expected, the corresponding peptide derived from the kinase-dead mutant lacked phosphorylation, confirming that the modification of Tyr171 arises from hCDKL5 autophosphorylation rather than from endogenous bacterial kinases. Beyond the TEY loop, additional phosphorylated residues (Ser343, Ser407, Ser481, Ser543, Ser720, and Ser847) were identified. These sites were all detected in TYP-grown samples, while Ser720 phosphorylation was also present in proteins produced under GG and GGM conditions.

Overall, these results demonstrate that growth medium composition strongly influences the phosphorylation landscape of recombinant hCDKL5. In particular, nutrient-rich conditions such as TYP result in both enhanced phosphorylation of the catalytic-loop tyrosine and a broader phosphorylation profile, which is likely to impact the activation state and enzymatic functionality of the protein (Table 3).

## 3. Discussion

Recent advances in therapeutic strategies for monogenic disorders have highlighted both the potential and the limitations of gene-based approaches, particularly for neurological diseases. Constraints related to vector capacity, long-term expression control, and immunogenicity continue to hamper their broad clinical translation, thereby sustaining interest in alternative strategies such as enzyme replacement therapy (ERT), which offers greater flexibility in dosing and protein engineering [43,44].

In this context, CDKL5 Deficiency Disorder (CDD) represents a paradigmatic case in which ERT has shown promising preclinical potential but remains limited by manufacturing challenges. Proof-of-concept studies demonstrated that systemic delivery of recombinant CDKL5 fused to a cell-penetrating peptide can rescue neurological defects in animal models [21], yet the lack of scalable and robust production platforms for full-length, biologically active CDKL5 has so far prevented clinical translation.

The recombinant production of hCDKL5 in the psychrophilic bacterium *Ph*TAC125 KrPl *LacY*^+^ therefore represents a significant step forward, confirming the suitability of this unconventional host for the expression of structurally complex and aggregation-prone human proteins [16]. This achievement builds upon extensive efforts aimed at developing *Ph*TAC125 as a microbial chassis through genome-scale metabolic modelling, medium optimisation, and tailored genetic tools [13,14,28]. Nevertheless, the production levels currently achievable remain insufficient for industrial application, underscoring the need for further process intensification.

Addressing this challenge requires a rational, process-oriented strategy that simultaneously considers yield, robustness, and product quality. In this study, we adopted a Quality by Design (QbD) framework to guide process development, emphasising the identification and control of critical quality attributes and process parameters from early stages [45]. Within this framework, the availability of sensitive and reliable analytical tools is essential. Accordingly, a major focus of this work was the development of a dedicated quantitative sandwich ELISA, designed to selectively detect full-length recombinant hCDKL5 and to overcome the intrinsic limitations of semi-quantitative approaches such as Western blot.

The assay design was intentionally aligned with the structural features of the recombinant construct, exploiting the presence of two terminal affinity tags. By targeting the N-terminal SUMO tag and the C-terminal 3xFLAG tag, the assay selectively quantified only the full-length recombinant protein, thereby excluding truncated or partially degraded species that would otherwise confound production estimates. This aspect is particularly relevant in the context of process development, where accurate discrimination between intact product and by-products is essential for meaningful optimisation. Within this framework, terminal tags serve as strategic enabling tools rather than defining features of quality, ensuring that the Quality by Design (QbD) framework remains centred on the protein’s intrinsic structural and functional attributes while providing a versatile analytical platform applicable to a wide range of other recombinant targets.

A key challenge in establishing a fully quantitative ELISA was the absence of a commercially available hCDKL5 standard. More generally, an effective standard for sandwich immunoassays must satisfy multiple requirements, including recognition by both antibodies, structural stability, ease of recombinant production, and suitability as a positive control. To address these constraints, a His-SUMO-eGFP-3xFLAG fusion protein was rationally designed. This construct preserves the immunological determinants required for assay recognition while leveraging the favourable biochemical properties of eGFP, a protein with well-documented recombinant production behaviour and a defined three-dimensional structure [46]. Importantly, the inclusion of eGFP as a structured spacer between the two epitopes was intended to minimise steric hindrance effects, thereby enhancing assay reliability [36].

The deliberate exclusion of an intrinsically disordered region (IDR) to mimic hCDKL5 architecture further reflects a process-oriented perspective. Although IDRs are characteristic of hCDKL5, their incorporation into the standard protein could have compromised expression yield and stability, ultimately undermining the robustness and reproducibility of the assay [24]. Following the successful production and validation of the standard, assay optimisation focused on defining critical analytical parameters, including capture and detection antibody concentrations and dilution buffer composition, in accordance with established ELISA development principles [33,37]. Lastly, the developed ELISA protocol requires a precise characterisation of the dose–response curve. Despite minor variations in the individual calibration equations, most replicates remained within the 95% confidence interval of the master average curve (Table A1). Nevertheless, the assay exhibits a narrow linear dynamic range (from 1 nM to 11 nM), necessitating the evaluation of multiple dilution factors for unknown samples to ensure accurate quantification. Despite this limitation, ELISA represents a more suitable analytical tool for protein quantification compared to Western blot (WB). Quantitative Western blot involves extensive sample manipulation, including SDS-PAGE, membrane transfer, and variable exposure times, which can introduce significant experimental bias [31]. Furthermore, while the ELISA protocol remains labour-intensive, it entirely bypasses the denaturing conditions required for electrophoresis, thereby allowing the monitoring of protein levels within soluble fractions [33,37]. Once the optimal working conditions of the ELISA had been established, the assay was applied to characterise recombinant hCDKL5 production in *Ph*TAC125 *KrPl LacY*^+^ grown under different culture conditions. The formulation of the GG synthetic medium is based on previous literature [47] and on genomic evidence indicating that *Ph*TAC125 primarily catabolises glucose through the Entner–Doudoroff pathway via its oxidation to gluconic acid [4]. The combination of D-Gluconate and L-Glutamate as carbon and energy sources was shown to efficiently sustain *Ph*TAC125 growth even at subzero temperatures, enabling the first reported recombinant protein production at −2.5 °C [9]. GG media, therefore, represented both the reference condition for recombinant protein expression in this host and the first medium used for hCDKL5 production [16].

Growth of *Ph*TAC125 in GG medium was mathematically described using a genome-scale stoichiometric metabolic model [8], which was further exploited by integrating experimental data on nutrient assimilation and hCDKL5 production to predict the global metabolic consequences of recombinant kinase expression and to guide rational medium optimisation [28]. The model accurately reproduced the phenotype of the recombinant strain and provided predictive indications on nutrient supplementation strategies, identifying key metabolic targets to enhance hCDKL5 overproduction. Among the nutrients suggested, maltose emerged as a potential intensifier capable of improving recombinant production when added to the GG medium.

In the present study, the performance of this metabolically optimised synthetic medium (GGM) was directly compared with that obtained using the GG medium. All key production parameters measured in GGM were higher than those observed in GG, thereby confirming the prediction generated by the in silico metabolic model [28]. Specifically, the model predicted that maltose supplementation would positively affect metabolic fluxes related to histidine and phenylalanine biosynthesis, as well as central sugar metabolism, ultimately increasing the flux directed towards recombinant hCDKL5 production. Our data validated these predictions and further showed that maltose supplementation also positively influenced biomass yield, growth rate, and overall productivity (Figure 5 and Table 2).

The third medium used in this work, the complex TYP medium, has been extensively tested with *Ph*TAC125 and is known to support robust bacterial growth and recombinant protein production, in agreement with previous reports describing the suitability of peptone-based media for this Antarctic marine bacterium [47]. As the IPTG-inducible psychrophilic promoter driving *hCDKL5* expression was approximately twofold less transcriptionally active in the TYP medium than in the GG medium [12], the recombinant protein production was prolonged till 24 h post-induction. This growth condition resulted in the highest biomass-associated Volumetric Yield and productivity, in line with previous observations that the Antarctic bacterium is adapted to rapid growth in nutrient-rich conditions and displays a highly flexible amino acid metabolism, relying on amino acids as central carbon and nitrogen resources [4]. Moreover, this difference in growth associated with the media tested is also reflected in the calculated biomass conversion factors. Cells recovered after growth in TYP presented a higher mg/OD_600nm_ ratio than those observed in synthetic media. Most probably, such a difference is related to the amount of nutrients available. Supporting this observation is the fact that GGM shows a higher conversion rate than the unmodified GG. With respect to recombinant hCDKL5 production, TYP medium enabled the recovery of the highest amount of protein per unit of processed biomass compared with the synthetic media, albeit over a longer production time (24 h). When production duration was considered, TYP and GGM resulted in comparable volumetric productivities of hCDKL5 (Table 2), extending and experimentally validating the insights provided by the metabolic modelling approach.

Phosphoproteomic analysis adds a crucial qualitative dimension to the evaluation of recombinant hCDKL5 production, both in absolute terms and across the different culture conditions examined. Importantly, the ELISA and phosphoproteomic assays provide complementary but not identical information. The former supports the presence of full-length hCDKL5, whereas the latter supports the presence of a phosphorylated and functionally competent hCDKL5 population. However, the present workflow does not formally demonstrate that the full-length species is the only phosphorylated species present in the sample. It should be noted that the enrichment and sample preparation workflow may introduce biases in the relative abundance of phosphorylated versus non-phosphorylated species. Therefore, the phosphorylation levels reported in this study should be interpreted as relative rather than absolute quantitative measures.

Because the bacterial host lacks endogenous human kinases capable of acting on CDKL5, all phosphorylation events detected in this system can be attributed to CDKL5 autophosphorylation (Table 3). This conclusion is further supported by LC–MS/MS analysis of the kinase-dead KK42–43RR mutant, in which no phosphorylation events were detected under the applied experimental conditions.

Consistent with this interpretation, among the multiple phosphorylation sites annotated for CDKL5 in the PhosphoSitePlus database (https://www.phosphosite.org/proteinAction.action?id=772&showAllSites=true access done on 2 February 2026), the residues S343, S407, S481, S543, S720, and S847 identified in this study can be ascribed to autophosphorylation events occurring within the intrinsically disordered region (IDR) of the enzyme. Although elucidating the biological significance of these auto-induced modifications lies beyond the scope of the present work, it is noteworthy that Ser720 is the only phosphoserine residue embedded within a near-canonical CDKL5 consensus motif (RPXS(A/P/G/S)). Moreover, Ser720 exhibited the highest phosphorylation occupancy among all identified sites, ranging from 7.9% to 33% across conditions, suggesting a preferential or more stable autophosphorylation event at this position. The broader presence of phosphorylated serine residues under nutrient-rich conditions may reflect enhanced kinase activity and/or improved protein stability and folding, both of which are critical determinants of functional quality in recombinant kinase production. Conversely, the limited detection of these sites in GG and GGM conditions suggests that nutrient constraints may restrict full post-translational maturation of hCDKL5.

In parallel, the phosphorylation of Tyr171 within the TEY activation loop a critical determinant of CDKL5 catalytic activity was detected under all culture conditions, albeit at markedly different levels, ranging from 0.5% to 1.9%. The systematic differences observed across media are consistent with genuine variations in enzyme activation rather than artefactual phosphorylation. Within this framework, the intermediate Tyr171 phosphorylation levels detected in GGM suggest that the metabolic optimisation of the synthetic GG medium partially supports hCDKL5 activation, in agreement with the concomitant improvements in production yield and biomass accumulation, while still falling short of the activation efficiency achieved under nutrient-rich TYP conditions.

To date, quantitative evaluations of Tyr171 phosphorylation have been reported only by our laboratory, comparing a CDKL5 catalytic fragment (1–352) produced in *E. coli* with a longer fragment (1–498) recombinantly expressed in insect cells and commercially available. In that study, only 0.8% of the bacterially produced catalytic domain was phosphorylated at Tyr171, whereas approximately 20% of the insect-cell-produced fragment carried this modification [25]. Considering that the long C-terminal IDR of CDKL5 has been shown to negatively regulate kinase activity [48], it is plausible that the presence of this regulatory tail may partially hinder Tyr171 autophosphorylation during protein synthesis, even in the Antarctic bacterial host. From this perspective, the 1.9% Tyr171 phosphorylation detected in hCDKL5 produced in TYP medium can be regarded as indicative of relatively high functional quality for a full-length recombinant enzyme.

Importantly, the strong dependence of Tyr171 phosphorylation on culture medium composition indicates that nutrient availability affects not only protein yield but also the functional maturation of the recombinant kinase. In particular, the higher Tyr171 phosphorylation levels observed in TYP-grown cultures compared with GGM and GG suggest that nutrient-rich conditions favour more efficient autophosphorylation and, consequently, a higher proportion of catalytically competent hCDKL5 molecules.

An additional factor that should be considered is the longer duration of the production process under TYP conditions, which may further contribute to increased Tyr171 phosphorylation by allowing more time for intra- and/or inter-molecular phosphorylation events. In contrast, extending induction in GG and GGM media to 24 h leads to CDKL5 predominantly accumulating in an insoluble form. Therefore, shorter induction times in GG and GGM are necessary to preserve solubility and functional activity (Supplementary Materials Figure S5). Nevertheless, the difference observed between GG and GGM, which were harvested within the same time window, indicates that medium composition per se also contributes to shaping the hCDKL5 phosphorylation profile. In this regard, studies on *Escherichia coli* grown in minimal medium have shown that median phosphorylation site occupancies are generally low throughout growth, but that a global increase in protein phosphorylation occurs approaching the stationary phase, suggesting a regulatory role for phosphorylation in later stages of bacterial physiology [49]. A similar growth-phase-dependent modulation of the phosphoproteome may also occur in *Ph*TAC125, potentially contributing to the enhanced phosphorylation profile observed in TYP-grown samples. However, a dedicated time-course analysis was not performed, and thus, the contribution of production time to the observed phosphorylation levels cannot be fully disentangled. Under this scenario, prolonged cultivation could favour the accumulation of phosphorylated hCDKL5 species, including Tyr171, independently or synergistically with nutrient availability. Future studies aimed at decoupling the effects of nutrient composition from growth phase—such as time-resolved phosphoproteomic analyses or controlled harvesting at defined physiological states—will be required to clarify the relative contribution of these parameters to kinase activation and functional quality.

## 4. Conclusions

Taken together, these results demonstrate that the combination of tailored analytical tools, model-guided medium design, and phosphoproteomic characterisation provides a powerful strategy to optimise both the quantity and functional quality of recombinant hCDKL5. Within a Quality by Design framework, the phosphorylation of Tyr171 and the associated phosphorylation profile emerge as critical quality attributes that should be considered alongside traditional productivity metrics. In this regard, TYP medium appears to provide a favourable balance between yield and functional activation, whereas GGM represents a partially effective, model-guided improvement over the GG baseline, consistent with predictions from the in silico metabolic analysis. Nevertheless, combining the current cultivation procedure of *Ph*TAC125 using a peptone-based rich medium at 15 °C with the induction of protein overexpression in a later exponential phase resulted in the optimal condition for the recombinant production of hCDKL5. More broadly, this work highlights the potential of psychrophilic bacterial platforms as viable hosts for the production of challenging human kinases and lays the groundwork for future studies aimed at further improving enzyme activation through the fine control of culture conditions and process timing.

## 5. Materials and Methods

### 5.1. xHis-SUMO-GFP-3xFlag Expression Vector Construction by Gibson Assembly

The pET28a-6xHis-SUMO-GFP-3xFlag vector was constructed by inserting the *egfp* gene into a pET28a vector between the SUMO and 3xFLAG tag sequences (Figure S1). The *egfp* sequence was PCR amplified using the *egfp*_fwd and *egfp*_rev primers and the *egfp_*insert gene fragment as a DNA template (Figure S3). The backbone was PCR amplified from the pET28a-6xHis-SUMO-3xFLAG plasmid using the pET28a-SUMO-3xFLAG_fwd and pET28a-SUMO-3xFLAG_rev primers (Figure S4). PCR amplifications were performed using Q5^®^ High-Fidelity DNA Polymerase (New England Biolabs (NEB), Ipswich, MA, USA, cat #M0491). All the primers used are listed in Table S1. All amplified fragments were purified using the PCR clean-up gel extraction kit (NEB) according to the manufacturer’s instructions. The PCR fragments and the vectors were assembled according to the one-step isothermal DNA assembly method using Gibson assembly (NEB, cat #E2611) described by Gibson et al. (2009) [50]. To validate that the assembly was successful, the expression vector was sequenced by Sanger sequencing. All DNA and amino acid sequences are available in the Supplementary Materials (Figures S2–S4, Tables S1 and S2, Supplementary3_egfp_gene_insert.fasta, Supplementary4_pET28a-6xHis-SUMO-3xFLAG.fasta and Supplementary5_pB40-BCD2-CDKL5(G7opt).fasta.). After incubation, the reaction was used to transform the *E. coli* TOP10 (Lab stock) competent cells by chemical transformation.

### 5.2. Culture Growth Conditions and Recombinant Expression

#### 5.2.1. *E. coli* BL21(DE3) Growth Condition and Standard Protein Recombinant Production

The pET28a-6xHis-SUMO-GFP-3xFLAG expression vector was transformed into *E. coli* BL21(DE3) (Lab stock, Naples, Italy) cell lines for recombinant production. The production was performed in 400 mL Lysogeny Broth (LB) [10 g/L Tryptone; 5 g/L yeast extract; 10 g/L NaCl] containing 50 μg/mL kanamycin in a 2 L Erlenmeyer flask sealed using foil cups. *E. coli* cells were grown at 37 °C, with 220 rpm agitation until a cellular concentration of 0.5 OD_600nm_/mL was reached. Hence, induction was performed with 0.05 mM isopropil-β-D-tiogalattopiranoside (IPTG). After induction, the cultures were incubated at 30°, at 220 rpm. A total of 5 OD_600nm_ cell pellets were harvested after 4 h post-induction. Cell pellets were centrifuged at 13,000 rpm at 4 °C for 10 min, then washed with PBS 1X and re-centrifuged. Pellets were stored at −20 °C.

#### 5.2.2. PhTAC125 KrPL LacY^+^ Growth Condition and hCDKL5 Recombinant Production

Recombinant production of hCDKL5 was performed in the *Ph*TAC125 KrPL *LacY^+^* strain harbouring the expression vector pB40-BCD2-CDKL5(G7opt) (Vector maps and sequence available in Supplementary Materials). Three media were tested for the recombinant production experiment: (i) a rich medium TYP (16 g/L Yeast-extract; 16 g/L Trypton; 10 g/L NaCl); (ii) a synthetic medium GG (5 g/L L-Glutamate; 5 g/L D-Gluconate); (iii) a synthetic medium called GG-maltose (5 g/L L-Glutamate; 5 g/L D-Gluconate; 10 g/L maltose). Both synthetic media were supplemented with salt Schatz (1 g/L K_2_HPO_4_; 10 g/L NaCl; 1 g/L NH_4_NO_3_; 200 mg/L MgSO_4_·7H_2_O; 5 mg/L FeSO_4_·7H_2_O; 5 mg/L CaCl_2_·2H_2_O).

For the rich medium, cells were streaked on solid TYP agar supplemented with 12.5 µg/mL chloramphenicol and incubated at 15 °C for four days. A single colony was inoculated in 3 mL of liquid TYP medium supplemented with 25 µg/mL chloramphenicol and incubated at 15 °C for two days. Regarding the TYP production condition, the pre-inoculum was diluted to 0.35 OD_600nm_/mL in 20 mL of TYP liquid media supplemented with 25 µg/mL chloramphenicol (100 mL Erlenmeyer flasks and foil cups were exploited as a sealing method) and incubated at 15 °C for 8 h with shaking. The inoculum was performed at 0.1 OD_600nm_/mL in 50 mL of TYP liquid media supplemented with 25 µg/mL chloramphenicol in a 250 mL Erlenmeyer flask sealed with foil cups. Cultures were incubated at 15 °C with shaking at 220 rpm overnight. Instead, when culturing cells using the synthetic media, an additional adaptation step was performed before the pre-inoculum. Specifically, cells were diluted to 0.2 OD_600nm_/mL in 10 mL of GG or GGM media supplemented with Schatz salts and 25 µg/mL chloramphenicol in a 100 mL Erlenmeyer flask sealed with foil cups, and incubated overnight at 15 °C with shaking at 220 rpm. Subsequently, the cultures were carried out in GG and GGM media using conditions as described for the TYP production. hCDKL5 recombinant production was induced with 5 mM IPTG when cells reached the middle–late exponential growth phase, corresponding to an OD6_600nm_ of approximately 2.0 for the TYP medium and an OD_600nm_ range of 1.3–2.6 for the synthetic media. Cells were collected at 24 h and 5 h post-induction, respectively, for the TYP medium and the synthetic media. Cell pellets were recovered by centrifugation at 4 °C, washed with PBS 1X, re-centrifuged, and stored at −20 °C. 

To determine the correlation factor between dry cell weight/OD_600nm_, sampling was performed during the exponential growth phase in three media. For each medium, a biomass volume corresponding to 7 OD_600nm_ units was collected by centrifugation at 5000 rpm for 10 min at 4 °C. The resulting pellets were washed with cold 1X PBS, re-centrifuged, and subsequently resuspended in PBS 1X. This suspension was then vacuum-filtered through 0.22 µm filters. The filters were dried in an oven overnight at 70 °C and weighed both before filtration (tare weight) and after drying to determine the dry biomass weight. Then the conversion factor mg to OD_600nm_ was calculated for each condition (Table 4).

Recombinant production of a kinase-dead hCDKL5 mutant (KK42–43RR variant) was carried out with *Ph*TAC125 KrPL *LacY*^+^ strain. Cells were grown in TYP medium, and the same growth protocol described for active hCDKL5 was followed.

### 5.3. E. coli BL21(DE3) Lysis and Standard Protein Purification

Cell pellets of 500 OD_600_ were resuspended in 30 mL of lysis buffer (25 mM NaPi, pH 7.2; 500 mM NaCl; 30 mM imidazole; 0.1 mg/mL lysozyme; 1X Roche Protease inhibitor complete Ultra EDTA free). Cell resuspension was subjected to two French-press cycles with a setting pressure of 2 kbar; then, 20 U/mL Dnase I and 1 mM MgCl_2_ were added to the lysate and incubated for 30 min at 4 °C. The soluble fraction was recovered by centrifugation at 6000 rpm for 45 min, then filtered using a 0.45 μm filter.

Purification of the standard protein was sorted out through two sequential purification steps: (i) immobilised metal affinity chromatography (IMAC) and (ii) anti-3xFLAG affinity chromatography (AF) (ANTI-FLAG^®^ M2, Merck, Darmstadt, Germany). The clarified *E. coli* lysate was loaded on a 5 mL IMAC column (GE Healthcare HisTrap HP, Uppsala, Sweden) connected to the AKTApure protein purification system (Cytiva, Uppsala, Sweden). Two wash steps of 7 column volumes (CVs) each were performed using 30 mM and 50 mM imidazole, respectively. Elution was performed in the following two steps. The first elution was carried out with 5 CV using 250 mM imidazole, while the second elution step was performed with 5 CV using 500 mM imidazole. The flow rate was set to 1 mL/min. The recovered fraction was subjected to dialysis using a 12 kDa cut-off membrane to remove imidazole. Three dialysis steps were performed at 4 °C in a dialysis buffer (25 mM NaPi, pH 7.2; 500 mM NaCl). After Bradford assay quantification, the dialysed fraction was loaded on a gravity column packed with 0.8 mL of the AF affinity resin. The resin was incubated overnight at 4 °C with tilting shaking. A wash step was performed using 15 CV of binding buffer (25 mM NaPi, pH 7.2; 500 mM NaCl), and then two elution steps of 2 CV each were performed using elution buffer (25 mM NaPi, pH 7.2; 500 mM NaCl; 0.5 mg/mL 3xFLAG peptide). Finally, glycerol was added to the eluted fraction to a final concentration of 20% *v*/*v* and stored at −80 °C.

Standard protein concentration was determined after the last purification step using the Bradford assay (Bio-Rad Protein Assay, Bio-Rad, Hercules, CA, USA), according to the manufacturer’s instructions.

### 5.4. SDS-PAGE and Western Blot Analysis

To assess protein expression levels, 2 OD_600_ cell pellets were resuspended in 140 μL of 4× Laemmli buffer and boiled at 95 °C for 20 min, rapidly cooled on ice for 1 min. In parallel, 2 OD_600_ cell pellets were lysed as reported by Colarusso et al. (2022) [16]. Then, the soluble fractions were collected by centrifugation, mixed with 4× Laemmli buffer, and denatured by boiling for 5 min. Total protein extracts and soluble fractions were separated by SDS–PAGE using 4–15% Mini-PROTEAN^®^ TGX™ precast gels (Bio-Rad, Hercules, CA, USA) and visualised by Coomassie Brilliant Blue staining. To detect hCDKL5, Western blot analysis was performed using the anti-CDKL5 primary antibody (Santa Cruz Biotechnology, CDKL5 (D-12), Santa Cruz, CA, USA, cat# sc-376314) as reported by Colarusso et al. (2022) [16].

### 5.5. Sandwich ELISA Protocol

The sandwich ELISA protocol described by Minic et al. (2020) [37] was used as a reference and then adapted to specific analytical requirements. The microplates used for this analysis were the MaxiSorp multiwell-96 (ThermoFisher Scientific™, Waltham, MA, USA, cat# 476635). The anti-SUMO capture antibody (GenScript, Piscataway, NJ, USA, cat# A01428-100) was diluted in PBS 1X to the desired concentration depending on the specific experiment. For the standard calibration curve and recombinant hCDKL5 quantification in bacterial lysate, the capture antibody was diluted to 0.2 μg/mL. A volume of 100 μL of capture antibody was added to each well, and the plate was left at 4 °C for 18 h. Subsequently, three wash steps were performed with 300 μL/well of PBST (PBS, 0.05% *v*/*v* Tween20) for 5 min. Subsequently, a blocking step was carried out with 300 μL of blocking buffer (PBST, 3% *w*/*v* BSA) and the plate was incubated at 37 °C for 2 h to avoid nonspecific binding to the well surface. Each well was treated. Three wash steps were performed with 300 μL/well of PBST for 5 min. Subsequently, the standard protein and soluble bacterial lysates were incubated on the plate for 18 h at 4 °C. All samples were diluted in 20 mM NaPi buffer (pH 7.2). The protein standard was tested at concentrations of 1.6, 0.8, 0.4, 0.2, 0.1, 0.05, and 0.025 µg/mL. Different dilution ranges were applied to *Ph*TAC125 lysates according to the specific growth media condition: dilutions ranging from 1:2 to 1:160 were analysed for TYP and GGM samples, while for the GG samples, a dilution ranging from 1:2 to 1:80 was tested. Each condition was analysed in technical triplicate. After incubation, three wash steps were carried out with 300 μL/well of PBST for 5 min. Following, the detection antibody (GenScript, anti-Flag HRP-conjugated, A01428-100) was diluted in blocking buffer to the desired concentration and incubated at 25 °C for 1 h. For the standard calibration curve and recombinant hCDKL5 quantification in bacterial lysate, the detection antibody was diluted to 0.15 μg/mL. Lastly, three wash-steps were carried out with 300 μL/well of PBST for 5 min. A total of 100 μL/well of TMB (3,3′,5,5′-Tetramethylbenzidine) solution was added to each well and incubated for a range of 15–20 min at room temperature, as a blue colouration was produced. The reaction was stopped with an equal volume of 0.18 M sulfuric acid. A colour shift from blue to yellow was observed, and the absorbance was detected at 450 nm using a spectrophotometer.

### 5.6. hCDKL5 Phosphorylation Characterisation by Mass Spectrometry Analysis

Enriched hCDKL5 protein preparations were carried out as reported by Colarusso et al. (2022) [16]. Cell lysis of recombinant *Ph*TAC125 cellular pellets was carried out using a chemical–enzymatic method. Then, the bacterial lysate was centrifuged to recover the soluble fraction. Clarified lysate was incubated with AF affinity gel to recover recombinant hCDKL5. Elution was performed with the 3xFLAG peptide [16]. The enriched fraction obtained from the different growth media cultivations was fractionated by SDS-PAGE and in situ hydrolysed as reported in [25,41,42]. Peptide mixtures were dissolved in 10 μL of 0.2% HCOOH (Merck Millipore, Burlington, MA, USA) and analysed by NanoLC-MS/MS on an Exploris 240 equipped with a nano-flow UHPLC system Vanquish™ Neo (ThermoFisher Scientific™, Waltham, MA, USA) and a Nanospray Flex™ion source (Waltham, USA). Samples were firstly concentrated onto a C18 capillary reverse-phase pre-column (ThermoFisher Scientific™ 5 mm, 300 μm, 5 μm), then fractionated on a C18 reversed-phase capillary column (ThermoFisher Scientific™, 150 mm, 75 μm, 2 μm 100 Å, Bellefonte, PA, USA) at an operating flow rate of 250 nl/min, employing a linear two-step gradient of buffer B (0.2% formic acid in 95% acetonitrile) in A (0.2% formic acid and 2% acetonitrile in LC-MS grade water). The MS/MS spectra were acquired in a Data-Dependent Acquisition (DDA) mode, consisting of a full scan in the range of 300–1800 *m*/*z* followed by the fragmentation of 30 ions for each MS scan, selected according to their intensities and charge states (+2, +3, and multiple charges) and considering a dynamic exclusion time window of 5 s. The output data were processed as reported by Colarusso et al. (2024) [25].

### 5.7. Statistical Analysis

The ELISA master curve was built by comparing 5 independent experiments, and regression analysis was performed using Microsoft Excel. Residual plot, R^2^ and 95% confidence range were estimated by such a tool. For LOD and LOQ estimations, the master curve angular coefficient (m) and standard deviation over intercept (s) were considered [38]. For hCDKL5 quantification, three independent replicas were compared. Data are reported as mean with the relative standard error of the measurement. Single-factor ANOVA analysis was applied to estimate *p*-values over each estimation. A *p*-value of <0.05 was considered statistically significant for all tests.

### 5.8. Mathematical Equations

To estimate assay LOD and LOQ, the residual method was applied. The following mathematical relations were used:

LOD = 3.3 × *s*/*m* (1)

LOQ = 10 × *s*/*m* (2)

where *s* is the standard deviation upon intercept, and *m* is the angular coefficient of the calibration curve [38].

[hCDKL5] is the estimated hCDKL5 molarity obtained from the ELISA quantification by means of the calibration curves (Appendix A.2). In this regard, the following formula was applied:

[hCDKL5] [nM]: (A_450nm_ − *q*)/*m* (3)

where *q* is the intercept of the equation, *m* is the angular coefficient, and A_450nm_ is the detected absorbance.

To estimate the maximum specific growth rate (µmax [h^−1^]), Biomass Volumetric Yield (Y_biomass_ [g·L^−1^]) and biomass volumetric productivity (Q_vbiomass_ [g·L^−1·^h^−1^]) biomass conversion factor of *Ph*TAC125 KrPl *LacY^+^* in each media, the following equations were used:

µmax = log_2_ (C2_bacterial_/C1_bacterial_)/(t2 − t1) (4)

Y_biomass_ = (m_cells_/V_growth_) (5)

Q_vbiomass_ = (Cf_bacteria_ − Ci_bacterial_)/(tf) (6)

The term C_bacterial_ refers to the bacterial concentration measured at different points of the growth curves, m_cells_ is the mass of cells recovered at the end of production, V_growth_ is the volume at which the growth was performed, and d and tx are the duration of the experiment expressed in hours (h). With regard to hCDKL5 production: hCDKL5 quantification (C_hCDKL5_ [µg/mL]), hCDKL5 Volumetric Yield (Y_hCDKL5_[g·L^−1^]), hCDKL5 specific content (%hCDKL5 [g·g^−1^]), hCDKL5 volumetric productivity (Q_VhCDKL5_[g·L^−1^·h^−1^]), and hCDKL5 Apparent Specific Yield (Ys_hCDKL5_ [mg/g]) were calculated using the following mathematical relations:

C_hCDKL_ = [hCDKL5] × MW_CDKL5_ (7)

Y_hCDKL5_ = (C_hCDKL5_.V_Lysis_)/(m_cells_/C_bacterial_) (8)

%hCDKL5 = m_hCDKL5_/m_totalproteincontent_ (9)

Q_VhCDKL5_ = (Y_hCDKL5_)/(tf) (10)

Ys_hCDKL5_ = m_hCDKL5measured_/m_cellsprocessed_ (11)

## Figures and Tables

**Table 1:** Growth kinetics and biomass-related parameters of the recombinant *Ph*TAC125 KrPl *LacY^+^* strain grown in different culture media.

Media	µ_max_^1^ (h^−1^) *	Final Biomass ^2^ (OD_600nm_) *	Biomass Volumetric Yield ^3^ (g·L^−1^) *	Biomass Volumetric Productivity ^4^ (g·L^−1^·h^−1^) *
TYP	0.19 ± 0.02	8.7 ± 1.3	7.7 ± 1.1	0.18 ± 0.04
GGM	0.19 ± 0.02	4.0 ± 1.1	2.7 ± 0.8	0.12 ± 0.03
GG	0.13 ± 0.01	2.4 ± 0.3	1.1 ± 0.1	0.05 ± 0.01

^1^ Maximum specific growth rate (µ_max_) estimated during the exponential phase. ^2^ Final biomass concentration reached at harvest, expressed as OD_600_. ^3^ Biomass Volumetric Yield expressed as g of cells ·L^−1^. ^4^ Biomass volumetric productivity expressed as Biomass Volumetric Yield divided by the duration of the production process (g·L^−1^·h^−1^). Data are reported as the mean ± standard deviation of three independent replicates. * *p* value ≤ 0.005.

**Table 2:** Production parameters of the recombinant expression of hCDKL5 with *Ph*TAC125 KrPl *LacY^+^* strain grown under different culture media.

Media	hCDKL5 Volumetric Yield (mg·L^−1^) ^1,^*	hCDKL5 Volumetric Productivity (mg·L^−1^·h^−1^) ^2,^*	hCDKL5 Phosphorylation Level of Tyr171 (%) ^3^
TYP	42.4 ± 8.8	1.0 ± 0.2	1.9
GGM	12.9 ± 4.6	0.6 ± 0.2	0.9
GG	1.6 ± 0.4	0.07 ± 0.03	0.5

^1^ hCDKL5 Volumetric Yield with *Ph*TAC125 KrPl *LacY^+^* strain. The parameter is expressed as mg of hCDKL5 recovered per L of *Ph*TAC125 KrPl *LacY^+^* culture broth processed. ^2^ hCDKL5 volumetric productivity expressed as hCDKL5 Volumetric Yield divided by the duration of the production process (mg·L^−1^·h^−1^). ^3^ % hCDKL5 phosphorylation level of TEY loop in the analysed production conditions. * *p*-value < 0.05.

**Table 3:** LC–MS/MS identification and quantification of hCDKL5 phosphopeptides.

CDKL5 Peptide	*m*/*z*	Sequence	Phospho-Site	GG (%)	GGM (%)	TYP (%)
159–175 * 159–175	998.4263 958.4451	NLSEGNNANYTE**Y**VATR	**Y171**	**0.5**	**0.9**	**1.9**
341–355 * 341–355	569.9548 543.2971	SN**S**KDIQNLSVGLPR	**S343**	n.d.	n.d.	**6.0**
396–409 * 396–409	552.6112 525.9555	DLTNNNIPHLL**S**PK	**S407**	n.d.	n.d.	**1.4**
479–483 * 479–483	345.1358 305.1532	SP**S**YR	**S481**	n.d.	n.d.	**1.4**
540–547 * 540–547	455.7212 415.7391	TLL**S**PSGR	**S543**	n.d.	n.d.	**2.2**
718–735 * 718–735	711.6562 685.0014	VP**S**PRPDNSFHENNVSTR	**S720**	**9.7**	**7.9**	**33.0**
842–858 * 842–858	623.6320 596.9733	LLHLS**S**ASNHPASSDPR	**S847**	n.d.	n.d.	**4.5**

Phosphorylated tryptic peptides identified in recombinant hCDKL5 produced in *Ph*TAC125 grown in TYP, GG, and GG supplemented with maltose (GGM) media. For each phosphopeptide, the table reports peptide start and end positions within the hCDKL5 sequence (the phosphorylated peptide is indicated by *), *m*/*z* values of the phosphorylated and corresponding non-phosphorylated forms, peptide sequence, the assigned phosphorylation site (in bold), and the relative phosphorylation percentage measured under each growth condition; n.d., not-detectable phosphorylation.

**Table 4:** Dry weight/OD_600nm_ conversion factor.

Media	Dry Weight/OD_600nm_ Conversion Factor
TYP	0.88 ± 0.11%
GGM	0.67 ± 0.0001%
GG	0.46 ± 0.005%

**Figure A1:**
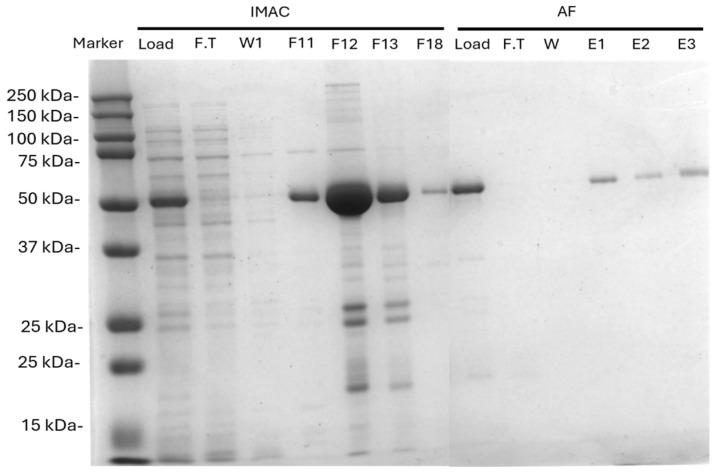
SDS-PAGE of standard 6xHis-SUMO-eGFP-3xFLAG purification. SDS-PAGE (12.5% polyacrylamide) images depict the two sequential purification steps, IMAC and AF affinity chromatography, from left to right, respectively. Legend: Load refers to the sampled loaded onto the column, the unbound fraction or Flow-Through (F.T), Wash fraction (W1), F refers to the fractions collected during the elution steps of the IMAC, and E refers to the elution fractions obtained during the elution step with anti-3xFLAG. Loaded volumes: Load, F.T, W1, W2 = 2 µL; F11, F12, F13, F18; E1,E2 = 1 µL.

## Data Availability

All data analysed during the current study are available from the corresponding authors upon reasonable request.

## Supplementary Materials

The following supporting information can be downloaded at: https://www.mdpi.com/article/10.3390/md24050151/s1. Figure S1: PhTAC125 KrPl LacY+ pB40-BDC2-CDKL5(G7opt) [12,16,25,39]. Growth curves in TYP(a), GG (b) and GGM media [9,28,47]. Cultures in the three media were grown at 15 °C with agitation at 220 rpm in 250 mL Erlenmeyer flasks [39]. Recombinant production was induced with 5 mM IPTG (red arrows). Cellular concentration was monitored by measuring absorbance at 600nm using a spectrophotometer. The growth curves are the average obtained from three inde-pendent replicas; Figure S2: ELISA Standard 6xHis-SUMO-GFP-3xFLAG expression vector map. The egfp sequence was cloned inside the vector pET28a-6xHis-SUMO-3xFlag, between the SUMO-tag and the 3xFLAG-tag by Gibson assembly method [36,46]. The map illustrates the genetic structure of the plasmid backbone: ori: origin of replication in E. coli strain; KanR: Kanamycin resistance gene; 6xHis-SUMO-GFP-3xFLAG construct; RBS. ribosome binding site; T7 promoter sequence; LacI gene. FASTA sequence in Supplementary2_pET28a-6xHis-SUMO-3xFLAG file; Figure S3: egfp gene insert sequence. The picture shows the egfp gene fragment inserted into the receiving vector (pET28a-6xHis-SUMO-3xFLAG) via Gibson assembly method for the construction of the ELISA standard protein [46]. The egfp gene insert fragment was amplified by PCR using Q5 polymerase and the primers egfp_fwd and egfp_rev. Template sequence reported in the Supplementary material as a FASTA file (Supplementary_egfp_gene_insert). In light blue, the linker (Sequence reported in Table S2) for was localised at 5′ of the sequence; meanwhile, a GS linker was added at the 3′. FASTA sequence in Supplementary3_egfp_gene insert file; Figure S4: Standard 6xHis-SUMO-3xFLAG expression vector map used as receiving vector for the Gibson assembly [50]. The receiving vector was amplified by PCR using the primers pET28a-SUMO-3xFLAG_fwd and pET28a-SUMO-3xFLAG_rev.The map illustrates the genetic structure of the plasmid backbone: ori: origin of replication in E. coli host; KanR: Kanamycin resistance gene; 6xHis-SUMO-3xFLAG construct; RBS: ribosome binding site; T7 promoter sequence; LacI gene. FASTA sequence in Supplementary4_pET28a-6xHis-SUMO-3xFLAG file; Figure S5: Solubility of hCDKL5 produced in PhTAC125 with GG and GGM media [12,16,28,39]. On the left the SDS-page coomassie stained, on the right Western blot analysis performed with the anti-CDKL5 antibody. The samples were collected 2, 4 and 24 h after the induction with 5mM IPTG. MW: Molecular weight; Table S1: Gibson assembly primers used for the cloning of the egfp sequence in the receiving vector pET28a-6xHis-SUMO-3Xflag [50]; Table S2 6xHis-SUMO-eGFP-3xFLAG and recombinant hCDKL5 amino acid sequence [21]; Supplementary2_pET28a-6xHis-SUMO-GFP-3xFLAG.fasta; Supplementary3_egfp_gene_insert.fasta; Supplementary4_pET28a-6xHis-SUMO-3xFLAG.fasta; Supplemen-tary5_pB40-BCD2-CDKL5(G7opt).fasta.

## Abbreviations

The following abbreviations are used in this manuscript:

AF	Anti-FLAG affinity chromatography
CDD	CDKL5 Deficiency Disorder
CV	Column volume
*E.coli*	*Escherichia coli*
ELISA	Enzyme-Linked Immunosorbent Assay
ERT	Enzyme replacement therapy
eGFP	Enhanced green fluorescent protein
GG	Gluconate–Glutamate medium
GGM	GG medium supplemented with maltose
hCDKL5	Human cyclin-dependent kinase-like 5
HRP	Horseradish peroxidase
IDR	Intrinsically disordered region
IMAC	Immobilised metal affinity chromatography
LC–MS/MS	Liquid chromatography–tandem mass spectrometry
LOD	Limit Of Detection
LOQ	Limit Of Quantification
NaPi	Sodium phosphate buffer
OD_600nm_	Optical density at 600 nm
PBST	Phosphate-buffered saline with Tween 20
PBS	Phosphate-buffered saline
PCR	Polymerase Chain Reaction
*Ph*TAC125	*Pseudoalteromonas Haloplanktis* TAC125
QbD	Quality by Design
SDS-PAGE	Sodium dodecyl sulphate–polyacrylamide gel electrophoresis
SUMO	Small ubiquitin-like modifier
TMB	3,3′,5,5′-tetramethylbenzidine
TS	Twin-Strep tag
TYP	Tryptone–yeast extract–peptone medium

## Appendix A

### Appendix A.1. Standard Protein Purification and ELISA Tests

Following recombinant production in *E. coli* (BL21), the standard protein 6xHis-SUMO-eGFP-3xFLAG (49 kDa) was purified via two sequential chromatographic steps, i.e., IMAC and Anti-3xFLAG affinity chromatography. Cell pellets were lysed by French-press, and the soluble fraction was loaded onto the IMAC to obtain an enriched preparation. Subsequently, the eluted fractions were loaded onto an anti-3xFLAG affinity gel to remove the undesired contaminants and recover the pure standard protein. Obtained fractions were analysed by SDS-PAGE to assess purity (Figure A1). The recombinant expression of 6xHis-SUMO-eGFP-3xFLAG was successful, as an overexpressed band at the expected molecular weight was clearly visible in the soluble bacterial extract (Load). The initial IMAC purification step was effective; nearly all of the target protein bound to the affinity column was subsequently recovered in three contiguous fractions (F11–F13). The second purification step using Anti-FLAG affinity gel successfully removed remaining contaminants, although the protein elution was sub-optimal as the product was diluted in the process. The titre obtained at the end of the purification process was 114 µg/mL with a purification yield of 70% of the 520 µg of standard protein, which was loaded on the AF column. Then, the standard protein was separately tested with the capture and detection antibodies intended for the sandwich ELISA. This step was key to confirming the correct exposure of the epitopes recognised by the two antibodies [36,37]. For both analyses, multiwell plates were derivatised with four different concentrations of standard protein (10.2 nM, 2.0 nM, 1.0 nM, and 0.4 nM). An indirect ELISA was carried out to assess capture antibody binding, and a direct ELISA was performed for the detection antibody (the results and the methodology are presented in Figure A2). The assays proved that both antibodies independently recognise the standard protein. Although both assays were carried out using equal antibody concentrations, indirect ELISA utilising the anti-SUMO antibody yielded higher output signals compared to direct ELISA used for the detection antibody. This difference may be attributed to the different ELISA designs applied; indirect ELISAs are characterised by higher sensitivity and signal amplification when compared to the direct format [32,33]. The next step consisted of analysing the ability of the two antibodies to recognise the standard protein in a sandwich configuration. The results are discussed in Section 2.2 of the main text (“Development of a sandwich ELISA for recombinant hCDKL5 analysis”). In Figure A3, the output data obtained with 10.2 nM and 0.4 nM are reported; the applied protocol is also described in the figure caption.

### Appendix A.2. ELISA Calibration Curves

After establishing the key assay conditions, the characterisation of the dose–response curve was performed. To this end, five independent experiments were conducted, and the resulting calibration curves were compared (Table A1). Data are reported in relation to the molar concentration (nM) of the standard protein. The calibration curves for Replicas 1 and 2 were constructed using two-fold dilutions ranging from 18 nM to 0.6 nM. To better characterise the linear dynamic range, a second set of three ELISA runs was performed, exploring dilutions from 60 nM to 1 nM. Within the explored range, the assays exhibited a linear response between 1 nM and 11 nM (0.05–0.53 μg/mL). For all the calibration curves obtained, minor variations in the slope (*m*) and intercept (*q*) were observed (Table A1). To assess the robustness of the calibration, these replicates were used to generate an average dose–response curve, followed by an analysis of the R^2^, residual distribution, and the *p*-value of the regression. These data are reported in Figure 3 and Figure A4. The resulting master curve presents an R^2^ of 0.96 and a statistically significant *p*-value (*p* < 0.05). Furthermore, the residual plot displays a scattered distribution around the *x*-axis, with no significant heteroscedasticity, bell-shaped trends, or outliers observed. In conclusion, while ELISA presents a narrow linear dynamic range, it maintains a strong correlation factor. For quantification purposes, the specific calibration curve from each run is applied, provided it falls within the 95% confidence interval of the average master curve.

**Table A1:** Calibration curves, equations, and 95% confidence range.

Calibration Curve	*m*	*q*	R^2^
DOSE–RESPONSE Master Curve	0.089 ± 0.008	0.0231 ± 0.047	0.96
Calibration Curve 1	0.09	0.049	0.99
Calibration Curve 2	0.08	−0.002	0.99
Calibration Curve 3	0.09	0.03	0.99
Calibration Curve 4	0.08	0.1	0.98
Calibration Curve 5	0.07	0.03	0.98

In the table, the angular coefficient (*m*), intercept (*q*), and R^2^ of the five calibration curve replicas are presented. On the first row, the equation for the average dose–response master curve is calculated from calibration curves 1, 2, 3, 4, and 5. The 95% confidence range for m and q is displayed in this column.

### Appendix A.3. CDKL5 Sample Analysis by ELISA

The biological matrix of interest, *Ph*TAC125 soluble lysates, was analysed. To evaluate whether endogenous molecules within the soluble fraction might cause interference, such as nonspecific binding, a sandwich ELISA was performed on non-induced (N.I.) samples from both TYP and GG media. On the same page, the overall mixture of the lysis buffer was analysed to assess the putative interferences of the signal detection. The data obtained (Table A2) demonstrate that no off-target products are recognised within the biological matrix in the absence of hCDKL5 recombinant production [36]. Indeed, the signals for all N.I. samples were comparable to the background signal obtained when plate wells were incubated only with the dilution buffer (20 mM NaPi, pH 7.2). The subsequent test consisted of assessing whether the buffer used could interfere with target protein identification via sandwich ELISA. The lysis buffer components were analysed to determine if they caused any signal interference. To this end, a spike-and-recovery assay was performed using the standard protein [36]. The lysis buffer was serially diluted with dilution buffer (undiluted, 1:2, and 1:10), and a known quantity of the standard was spiked to a final concentration of 0.5 μg/mL (Table A3). The signals obtained were compared to a reference condition where the standard protein was spiked directly into the dilution buffer. Significant interference was detected in the undiluted lysis buffer, likely due to specific components that interfere with the recognition of the standard protein by the antibodies. This interference was effectively mitigated by diluting the lysis buffer by a factor of 1:10 before adding the standard. Consequently, to overcome this interference and ensure the samples remained within the narrow linear range of the assay, all biological samples were diluted accordingly. A dilution range that goes from 1:10 to 1:160 was tested for all samples. Dilution points in which the ELISA signal was reduced accordingly were used for quantification by means of the calibration curve. Using this optimised protocol, samples recovered after hCDKL5 expression in *Ph*TAC125 across the three media (TYP, GG, and GGM) were analysed. Detailed results are discussed in Section 2.3 of the main text, and Figure A4 displays the calibration curves obtained for the three replicates. Although the results are reported in mass units (μg/mL), due to the difference in molecular weight between the standard protein (49 kDa) and the target molecule hCDKL5 (129 kDa), quantification was first performed considering molar concentrations and then converted to mass units. In this way, differences due to the distinct sizes of the proteins were accounted for in the quantification.

**Table A2:** Background noise and interference in biological matrix.

Sample ^1^	Absorbance 450nm ^2^
Dilution buffer	0.053 ±0.002
N.I TYP	0.046 ± 0.007
N.I GG	0.046 ± 0.002
Lysis buffer	0.057 ± 0.001

^1^ Analysed samples: Samples were analysed by means of a sandwich ELISA to assess interference in the detection signal. Dilution buffer: 20 mM NaPi, pH 7.2.; N.I. TYP: Bacterial lysate obtained by processing samples recovered from *Ph*TAC125 in TYP medium without IPTG induction of recombinant production; N.I. GG: Bacterial lysate obtained by processing samples recovered from *Ph*TAC125 in GG medium without IPTG induction of recombinant production; lysis buffer exploited for the bacterial lysis and soluble fraction extraction (Materials and Methods Section). ^2^ Absorbance at 450 nm: The output signal obtained at 450 nm at the end of the assay for each condition is reported.

**Table A3:** Spike-and-recovery test to assess sample interference.

Sample ^1^	Absorbance 450 nm ^2^
Dilution buffer	1.05 ± 0.03
Lysis buffer (not diluted)	0.03 ± 0.03
Lysis buffer (1:2)	0.15 ± 0.03
Lysis buffer (1:10)	0.80 ± 0.04

^1^ Analysed samples: Samples were analysed by means of a sandwich ELISA to assess interference in the standard protein recognition; dilution buffer: 20 mM NaPi, pH 7.2; lysis buffer: spike-and-recovery test carried out using the lysis buffer; 1:2 and 1:10 dilutions were carried out in the dilution buffer. In all samples, standard protein was spiked at a concentration of 0.5 μg/mL. ^2^ Absorbance at 450 nm: The output signal obtained at 450 nm at the end of the assay for each condition is reported.
