# Supplementary material for: From Sea to Therapy: Development and Analytical Control of Recombinant Human CDKL5 Production in the Marine Bacterium Pseudoalteromonas haloplanktis TAC125
Source: Mar Drugs. 2026 Apr 24;24(5):151. doi: 10.3390/md24050151 (PMC13208570; doi:10.3390/md24050151)
Supplement: Supplementary file 1 [file marinedrugs-24-00151-s001.zip › marinedrugs-4172747-supplementary 1.pdf]

marine drugs

Article

From Sea to Therapy: Development and analytical control of recombinant human CDKL5 production in the marine bacterium P*seudoalteromonas haloplanktis* TAC125

Coletti Andrea[1], Calvanese Marzia [1,2,*], Cozzolino Flora [1,3], Iacobucci Ilaria [1,3], Lauro Concetta [1], Severino Angelica [1,2], Monti Maria [1,3], Parrilli Ermenegilda [1] and Maria Luisa Tutino [1,2,*]

[1] Department of Chemical Sciences, University of Naples Federico II, Complesso Universitario Monte S. Angelo, Via Cintia 4, 80126 Naples, Italy; andrea.coletti@unina.it (C.A.); flora.cozzolino@unina.it (C.F.); ilaria.iacobucci@unina.it (I.I.); concetta.lauro@unina.it (L.C.); angelica.severino@unina.it (S.A.); montimar@unina.it (M.M.); ermenegilda.parrilli@unina.it (P.E.)
[2] Istituto Nazionale Biostrutture e Biosistemi I.N.B.B., Via dei Carpegna, 19, 00165 Roma, Italy
[3] CEINGE Advanced Biotechnologies, Via Gaetano Salvatore 486, 80145 Naples, Italy
* Correspondence: marzia.calvanese@unina.it (C.M.); tutino@unina.it (M.L.T.)

Academic Editor(s): Name

Received: 9 February 2026
Revised: 11 April 2026
Accepted: 20 April 2026
Published: date

Copyright: © 2026 by the authors. Submitted for possible open access publication under the terms and conditions of the Creative Commons Attribution (CC BY) license.

Supplementary

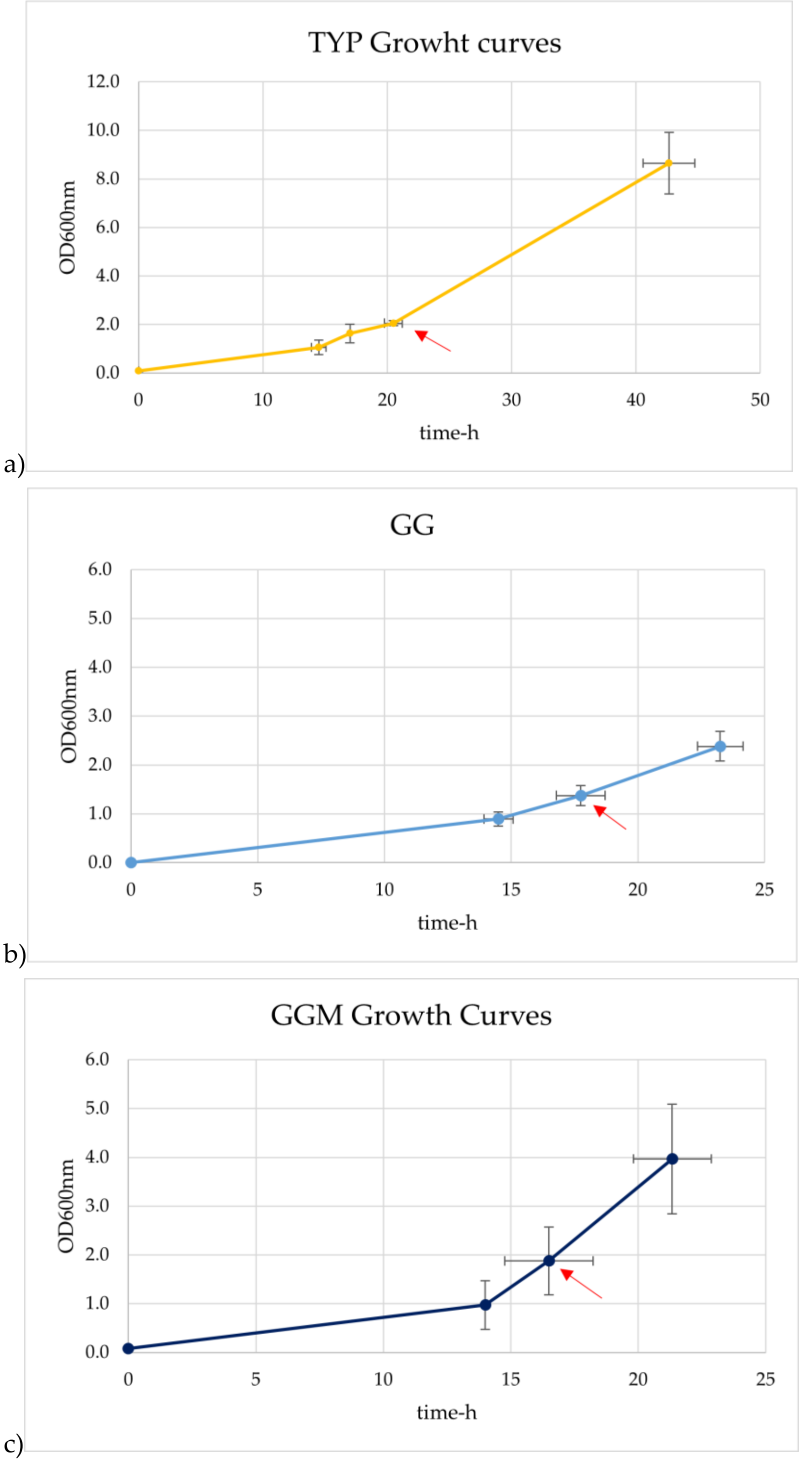

Figure S1. *Ph*TAC125 KrPl *LacY*⁺ pB40-BDC2-CDKL5(G7opt)[1–4]. Growth curves in TYP(a), GG (b) and GGM media [5–7]. Cultures in the three media were grown at 15 °C with agitation at 220 rpm in 250 mL Erlenmeyer flasks [4]. Recombinant production was induced with 5 mM IPTG (red arrows). Cellular concentration was monitored by measuring absorbance at 600nm using a spectrophotometer. The growth curves are the average obtained from three independent replicas.

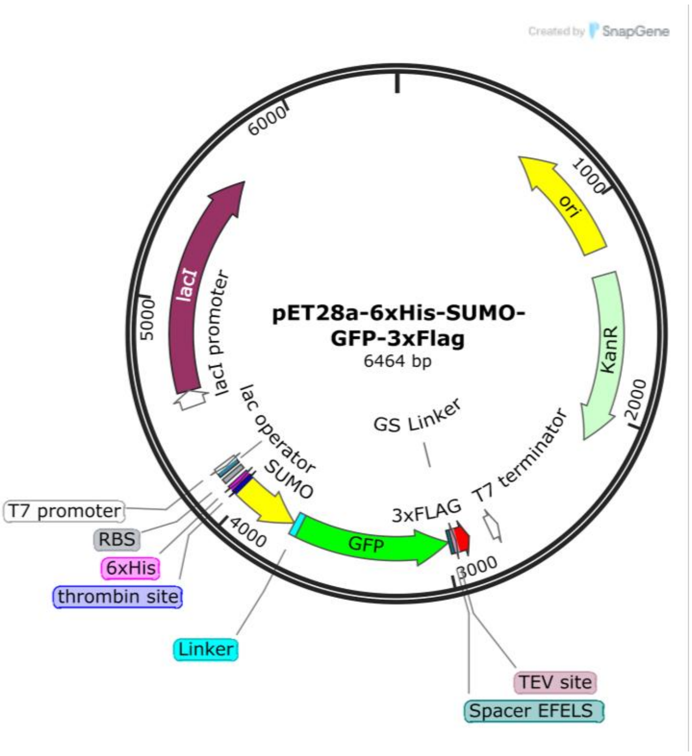

Figure S2. ELISA Standard 6xHis-SUMO-GFP-3xFLAG expression vector map. The *egfp* sequence was cloned inside the vector pET28a-6xHis-SUMO-3xFlag, between the SUMO-tag and the 3xFLAG-tag by Gibson assembly method [8,9]. The map illustrates the genetic structure of the plasmid backbone: ori: origin of replication in *E. coli* strain; KanR: Kanamycin resistance gene; 6xHis-SUMO-GFP-3xFLAG construct; RBS. ribosome binding site; T7 promoter sequence; LacI gene. FASTA sequence in Supplementary2_pET28a-6xHis-SUMO-3xFLAG file.

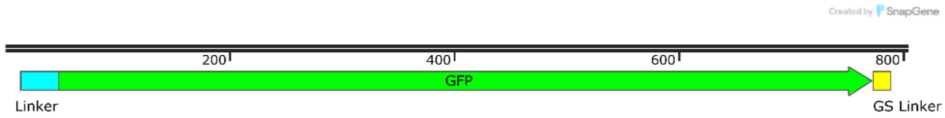

Figure S3. *egfp gene* insert sequence. The picture shows the *egfp* gene fragment inserted into the receiving vector (pET28a-6xHis-SUMO-3xFLAG) via Gibson assembly method for the construction of the ELISA standard protein [9]. The *egfp gene* insert fragment was amplified by PCR using Q5 polymerase and the primers *egfp*_fwd and *egfp*_rev. Template sequence reported in the Supplementary material as a FASTA file (*Supplementary_egfp_gene_insert*). In light blue, the linker (Sequence reported in Table S.2) for was localised at 5′of the sequence; meanwhile, a GS linker was added at the 3′ . FASTA sequence in Supplementary3_egfp_gene insert file.

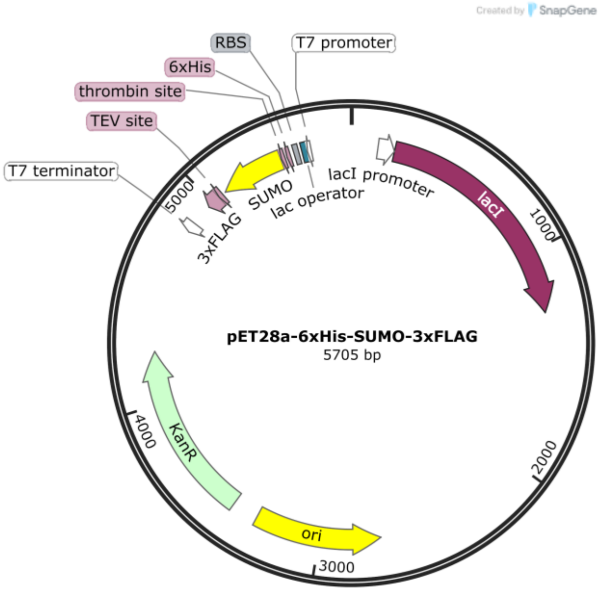

Figure S.4 Standard 6xHis-SUMO-3xFLAG expression vector map used as receiving vector for the Gibson assembly [10]. The receiving vector was amplified by PCR using the primers pET28a-SUMO-3xFLAG_fwd and pET28a-SUMO-3xFLAG_rev.The map illustrates the genetic structure of the plasmid backbone: ori: origin of replication in *E. coli* host; KanR: Kanamycin resistance gene; 6xHis-SUMO-3xFLAG construct; RBS: ribosome binding site; T7 promoter sequence; LacI gene. FASTA sequence in Supplementary4_pET28a-6xHis-SUMO-3xFLAG file.

Table S.1 Gibson Assembly Primers used for the cloning of the pET28a-6xHis-SUMO-3xFLAG vector[10].

Primers	Sequence
*egfp*_fwd	GTGGATCCCCAGCCGGGGCTCTGATTAA
*egfp*_rev	CTCGAATTCTTTGTAGAGCTCATCCATGCCATGTG
pET28a-SUMO-3xFLAG_fwd	CGGCTGGGGATCCACCAATCTGTTCACG
pET28a-SUMO-3xFLAG_rev	GCTCTACAAAGAATTCGAGCTCTCTGAGAATTTATA TTTCCAAGGTG

Primers were designed to clone the *egfp* sequence into the pET28a-6xHis-SUMO-3xFLAG vector via Gibson Assembly.

Table S.2 6xHis-SUMO-eGFP-3xFLAG and recombinant hCDKL5 amino acid sequence.

Protein	Amino acid sequence
**6xHis-SUMO-eGFP-3xFLAG 49kDa**	MGSSHHHHHHSSGLVPRGSHMGSDSEVNQEAKPEVKPEVKPETHIN<br>LKVSDGSSEIFFKIKKTTPLRRLMEAFAKRQGKEMDSLRFLYDGIRIQA<br>DQTPEDLDMEDNDIIEAHREQIGGSPAGALINMHGTGGGNSSKGEE<br>LFTGVVPILVELDGDVNGHKFSVSGEGEGDATYGKLTLKFICTTGKLPV<br>PWPTLVTTLTYGVQCFSRYPDHMKRHDFFKSAMPEGYVQERTISFKD<br>DGNYKTRAEVKFEGDTLVNRIELKGIDFKEDGNILGHKLEYNYNSHNV<br>YITADKQKNGIKANFKIRHNIEDGSVQLADHYQQNTPIGDGPVLLPDN<br>HYLSTQSALSKDPNEKRDHMVLLEFVTAAGITHGMDELYKEFELSENL<br>YFQGDYKDHDGDYKDHDIDYKDDDDK
**Recombinant hCDKL5 129kDa**	MASAWSHPQFEKGGGSGGGSGGSAWSHPQFEKGSDSEVNQEAKP<br>EVKPEVKPETHINLKVSDGSSEIFFKIKKTTPLRRLMEAFAKRQGKEMD<br>SLRFLYDGIRIQADQTPEDLDMEDNDIIEAHREQIGGMGDAAQPARR<br>ARRTKLAAYARKAARQARAGGGGSKIPNIGNVMNKFEILGVVGEGAY<br>GVVLKCRHKETHEIVAIKKFKDSEENEEVKETTLRELKMLRTLKQENIVE<br>LKEAFRRRGKLYLVFEYVEKNMLELLEEMPNGVPPEKVKSYIYQLIKAI<br>HWCHKNDIVHRDIKPENLLISHNDVLKLCDFGFARNLSEGNNANYTE<br>YVATRWYRSPELLLGAPYGKSVDMWSVGCILGELSDGQPLFPGESEID<br>QLFTIQKVLGPLPSEQMKLFYSNPRFHGLRFPAVNHPQSLERRYLGILN<br>SVLLDLMKNLLKLDPADRYLTEQCLNHPTFQTQRLLDRSPSRSAKRKP<br>YHVESSTLSNRNQAGKSTALQSHHRSNSKDIQNLSVGLPRADEGLPA<br>NESFLNGNLAGASLSPLHTKTYQASSQPGSTSKDLTNNNIPHLLSPKEA<br>KSKTEFDFNIDPKPSEGPGTKYLKSNSRSQQNRHSFMESSQSKAGTLQ<br>PNEKQSRHSYIDTIPQSSRSPSYRTKAKSHGALSDSKSVSNLSEARAQIA<br>EPSTSRYFPSSCLDLNSPTSPTPTRHSDTRTLLSPSGRNNRNEGTLDSR<br>RTTTRHSKTMEELKLPEHMDSSHSHSLSAPHESFSYGLGYTSPFSSQQ<br>RPHRHSMYVTRDKVRAKGLDGSLSIGQGMAARANSLQLLSPQPGEQ<br>LPPEMTVARSSVKETSREGTSSFHTRQKSEGGVYHDPHSDDGTAPKE<br>NRHLYNDPVPRRVGSFYRVPSPRPDNSFHENNVSTRVSSLPSESSSGT<br>NHSKRQPAFDPWKSPENISHSEQLKEKEKQGFFRSMKKKKKSQTVP<br>NSDSPDLLTLQKSIHSASTPSSRPKEWRPEKISDLQTQSQPLKSLRKLLH<br>LSSASNHPASSDPRFQPLTAQQTKNSFSEIRIHPLSQASGGSSNIRQEP<br>APKGRPALQLPGQMDPGWHVSSVTRSATEGPSYSEQLGAKSGPNG<br>HPYNRTNRSRMPNLNDLKETALGGGGSENLYFQGDYKDHDGDYKD<br>HDIDYKDDDDK

The full amino acid sequence of the Standard protein and hCDKL5 are displayed. For 6xHis-SUMO-eGFP-3xFLAG, the following schematisation is used: 6xHis-tag is represented in dark grey, thrombine binding site in pink, SUMO-tag sequence in light blue, eGFP protein sequence in yellow, TEV protease binding site in purple and 3xFLAG-tag in red. For the hCDKL5 protein sequence, the following schematisation is used: TwinStrep-Tag is represented in light grey, SUMO-tag sequence in light blue, TATk signal peptide sequence is dark blue [11], recombinant hCDKL5 sequence is represented in orange (Catalytic domain is represented in orange. IDR domain is represented in orange), TEV protease binding site in purple and 3xFLAG-tag in red. All speaker sequences are left in black.

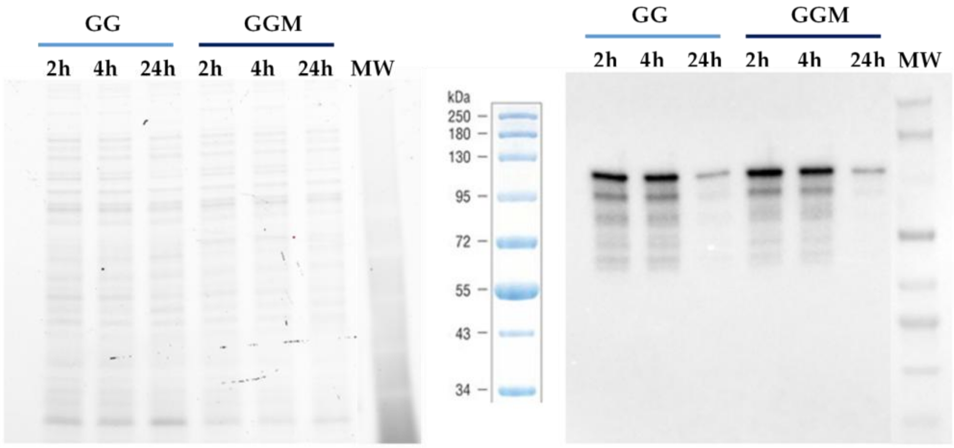

Figure S.5 Solubility of hCDKL5 produced in *Ph*TAC125 with GG and GGM media [1,2,4,5]. On the left the SDS-page coomassie stained, on the right Western blot analysis performed with the anti-CDKL5 antibody. The samples were collected 2, 4 and 24 hours after the induction with 5mM IPTG. MW: Molecular weight.

References

1. Colarusso, A.; Lauro, C.; Calvanese, M.; Parrilli, E.; Tutino, M.L. Active Human Full-Length CDKL5 Produced in the Antarctic Bacterium Pseudoalteromonas Haloplanktis TAC125. *Microb. Cell Fact.* **2022**, *21*, doi:10.1186/s12934-022-01939-6.
2. Colarusso, A.; Lauro, C.; Calvanese, M.; Parrilli, E.; Tutino, M.L. Improvement of Pseudoalteromonas Haloplanktis Tac125 as a Cell Factory: Iptg-Inducible Plasmid Construction and Strain Engineering. *Microorganisms* **2020**, *8*, 1–24, doi:10.3390/microorganisms8101466.
3. Colarusso, A.; Lauro, C.; Canè, L.; Cozzolino, F.; Tutino, M.L. Bacterial Production of CDKL5 Catalytic Domain: Insights into Aggregation, Internal Translation and Phosphorylation Patterns. *Int. J. Mol. Sci.* **2024**, *25*, doi:10.3390/ijms25168891.
4. Calvanese, M.; Colarusso, A.; Lauro, C.; Parrilli, E.; Tutino, M.L. Soluble Recombinant Protein Production in Pseudoalteromonas Haloplanktis TAC125: The Case Study of the Full-Length Human CDKL5 Protein. In *Methods in Molecular Biology*; Humana Press Inc., Totowa, NJ, USA 2022; Vol. 2406, pp. 219–232.
5. Fondi, M.; Gonzi, S.; Dziurzynski, M.; Turano, P.; Ghini, V.; Calvanese, M.; Colarusso, A.; Lauro, C.; Parrilli, E.; Tutino, M.L. Modelling HCDKL5 Heterologous Expression in Bacteria. *Metabolites* **2021**, *11*, doi:10.3390/metabo11080491.
6. Sannino, F.; Giuliani, M.; Salvatore, U.; Apuzzo, G.A.; de Pascale, D.; Fani, R.; Fondi, M.; Marino, G.; Tutino, M.L.; Parrilli, E. A Novel Synthetic Medium and Expression System for Subzero Growth and Recombinant Protein Production in Pseudoalteromonas Haloplanktis TAC125. *Appl. Microbiol. Biotechnol.* **2017**, *101*, 725–734, doi:10.1007/s00253-016-7942-5.
7. Wilmes, B.; Hartung, A.; Lalk, M.; Liebeke, M.; Schweder, T.; Neubauer, P. *Fed-Batch Process for the Psychrotolerant Marine Bacterium Pseudoalteromonas Haloplanktis*; *Microb. Cell Factories* **2010**, *9*, 72.
8. Wild David *The Immunoassay Handbook Theory and Applications of Ligand Binding, ELISA and Related Techniques*; Fourth Edition.; 2013;
9. March, J.C.; Rao, G.; Bentley, W.E. Biotechnological Applications of Green Fluorescent Protein. *Appl. Microbiol. Biotechnol.* 2003, *62*, 303–315.

10. Gibson, D.G.; Young, L.; Chuang, R.Y.; Venter, J.C.; Hutchison, C.A.; Smith, H.O. Enzymatic Assembly of DNA Molecules up to Several Hundred Kilobases. *Nat. Methods* **2009**, *6*, 343–345, doi:10.1038/nmeth.1318.
11. Trazzi, S.; De Franceschi, M.; Fuchs, C.; Bastianini, S.; Viggiano, R.; Lupori, L.; Mazziotti, R.; Medici, G.; Martire, V. Lo; Ren, E.; et al. CDKL5 Protein Substitution Therapy Rescues Neurological Phenotypes of a Mouse Model of CDKL5 Disorder. *Hum. Mol. Genet.* **2018**, *27*, 1572–1592, doi:10.1093/hmg/ddy064.

Disclaimer/Publisher's Note: The statements, opinions and data contained in all publications are solely those of the individual author(s) and contributor(s) and not of MDPI and/or the editor(s). MDPI and/or the editor(s) disclaim responsibility for any injury to people or property resulting from any ideas, methods, instructions or products referred to in the content.
